# Aromatic compounds depurative and plant growth promotion rhizobacteria abilities of *Allenrolfea vaginata* (*Amaranthaceae*) rhizosphere microbial communities from a solar saltern hypersaline soil

**DOI:** 10.3389/fmicb.2023.1251602

**Published:** 2023-10-23

**Authors:** Gustavo Rodríguez-Valdecantos, Felipe Torres-Rojas, Sofía Muñoz-Echeverría, Merit del Rocío Mora-Ruiz, Ramon Rosselló-Móra, Luis Cid-Cid, Thomas Ledger, Bernardo González

**Affiliations:** ^1^Facultad de Ingeniería y Ciencias, Universidad Adolfo Ibáñez, Santiago, Chile; ^2^Center of Applied Ecology and Sustainability (CAPES), Santiago, Chile; ^3^Marine Microbiology Group, Department of Animal and Microbial Biodiversity, Mediterranean Institute for Advanced Studies (IMEDEA UIB-CSIC), Esporles, Spain

**Keywords:** aromatic compounds, halophiles, PGPR, rhizosphere, salterns

## Abstract

**Introduction:**

This work investigates whether rhizosphere microorganisms that colonize halophyte plants thriving in saline habitats can tolerate salinity and provide beneficial effects to their hosts, protecting them from environmental stresses, such as aromatic compound (AC) pollution.

**Methods:**

To address this question, we conducted a series of experiments. First, we evaluated the effects of phenol, tyrosine, 4-hydroxybenzoic acid, and 2,4-dichlorophenoxyacetic (2,4-D) acids on the soil rhizosphere microbial community associated with the halophyte *Allenrolfea vaginata*. We then determined the ability of bacterial isolates from these microbial communities to utilize these ACs as carbon sources. Finally, we assessed their ability to promote plant growth under saline conditions.

**Results:**

Our study revealed that each AC had a different impact on the structure and alpha and beta diversity of the halophyte bacterial (but not archaeal) communities. Notably, 2,4-D and phenol, to a lesser degree, had the most substantial decreasing effects. The removal of ACs by the rhizosphere community varied from 15% (2,4-D) to 100% (the other three ACs), depending on the concentration. *Halomonas* isolates were the most abundant and diverse strains capable of degrading the ACs, with strains of *Marinobacter*, *Alkalihalobacillus*, *Thalassobacillus*, *Oceanobacillus*, and the archaea *Haladaptatus* also exhibiting catabolic properties. Moreover, our study found that halophile strains Halomonas sp. LV-8T and *Marinobacter* sp. LV-48T enhanced the growth and protection of *Arabidopsis thaliana* plants by 30% to 55% under salt-stress conditions.

**Discussion:**

These results suggest that moderate halophile microbial communities may protect halophytes from salinity and potential adverse effects of aromatic compounds through depurative processes.

## Introduction

Aromatic compounds (AC) derived from agro-industrial activities may contaminate saline and hypersaline environments (Fathepure, 2014). Most of these compounds have impacts on animals (including humans), plants, and environmental health because they are toxic, mutagenic, and carcinogenic (Honda and Suzuki, 2020; Krzyszczak and Czech, 2021; Molina and Segura, 2021). Although many microorganisms can degrade AC through both anaerobic and, most frequently, aerobic pathways, cleaving the aromatic ring into Krebs’s cycle intermediate metabolism (Pérez-Pantoja et al., 2010a), it is clear that salinity is one of the main factors that affect AC degradation performance (Li et al., 2019; Mainka et al., 2021). However, halophilic microorganisms from diverse saline environments possess AC degradation capabilities (Bonfá et al., 2013; Li et al., 2019). Moderate halophiles have thus been used to remediate contamination by AC at mild saline concentrations (Ma et al., 2010; Veenagayathri and Vasudevan, 2015; Guo et al., 2016). Some extreme halophiles have also been recommended for bioremediation of saline environments polluted with AC (Arora et al., 2017; Aracil-Gisbert et al., 2018). The application of whole halophilic microbial communities or consortia to degrade xenobiotic compounds under saline and hypersaline conditions has also been proposed (Aracil-Gisbert et al., 2018; Kimbrel et al., 2018).

Halophilic microorganisms that degrade AC belong to different taxa; however, the *Pseudomonadota* family *Halomonadaceae*, which mainly comprises marine and moderate halophiles, has been identified as the most diverse group able to degrade phenol and phenol by-products under moderately halophilic conditions (Bonfá et al., 2013; Al Farraj et al., 2020). In addition, species belonging to *Bacillaceae* (phylum *Bacillota*), *Micrococcaceae*, *Nocardiaceae*, and *Dermabacteraceae* (phylum *Actinomycetota*) have also shown potential for bioremediation of organic pollutants (Li et al., 2019). All these halophiles may thrive in environments contaminated by AC (Álvarez and Silva, 2013). Some of these halophilic AC degrading groups have also been associated with plant growth promotion (Mora-Ruiz et al., 2015; Marasco et al., 2016; Sáenz-Mata et al., 2016). Other plant growth promotion rhizobacteria (PGPR) and microbial consortia could degrade organic pollutants found in soil and make plants more tolerant to such abiotic stressors (Chaudhary et al., 2023). Some PGPR strains can protect plants from saline stress (Pinedo et al., 2015). Such beneficial plant-bacteria interactions may be affected by AC (Sampedro et al., 2020). Therefore, identifying and studying halophilic rhizosphere microorganisms able to degrade AC is essential to increase the understanding of how the halophilic microbial community can enhance plant growth and exhibit health protection capacities.

*Allenrolfea vaginata,* belonging to the family *Amaranthaceae,* subfamily *Salicornioideae* (Mora-Ruiz et al., 2015), represents a small group of halophytes that play an essential role in the preservation of coastal ecosystems due to their high salt tolerance and their specialized associated microbiota (Jafari et al., 2012). Salinity-tolerant rhizosphere microorganisms associated with *Amaranthaceae* species have an essential role in enhancing growth and salt tolerance in this halophyte (Jafari et al., 2012; Mora-Ruiz et al., 2015), and some of these microorganisms have been proposed as biofertilizers for improving the growth of the host-plant in saline environments (Jafari et al., 2012; Mora-Ruiz et al., 2015; Razzaghi Komaresofla et al., 2019). It is worth noting that tolerance to extreme saline conditions varies from one plant species to another, and the impact level of such abiotic stress on growth and development is species-dependent (Sardar et al., 2023). Advances have also been described in the use of halophiles to protect and promote the growth of non-halophyte commercial crops such as tomatoes (Sahu et al., 2023) and rice (Mallick et al., 2018; Kapadia et al., 2022). However, information about rhizosphere microorganisms colonizing halophytes and non-halophytes and their biotechnological potential is still scarce (Sáenz-Mata et al., 2016; Mahmood et al., 2019).

To contribute to the knowledge of catabolic and plant growth promotion abilities of *A. vaginata*-associated microbiota, the effects on the *A. vaginata* rhizosphere bacterial and archaeal communities of four low molecular weight AC are reported here. The isolation and initial characterization of microorganisms removing these AC and their promoting plant growth properties under saline stress are also described. 2,4-dichlorophenoxyacetic acid (2,4-D), 4-hydroxybenzoic acid (4-HB), phenol (Phe), and tyrosine (Tyr) were chosen as these four ACs are commonly found in industrial wastewater and have been considered chemical models to study pollution effects and biodegradation capabilities (Pérez-Pantoja et al., 2008; Ning et al., 2015; Warren-Vega et al., 2023).

## Materials and methods

### Soil samples and microcosms set up

**Figure 1:**
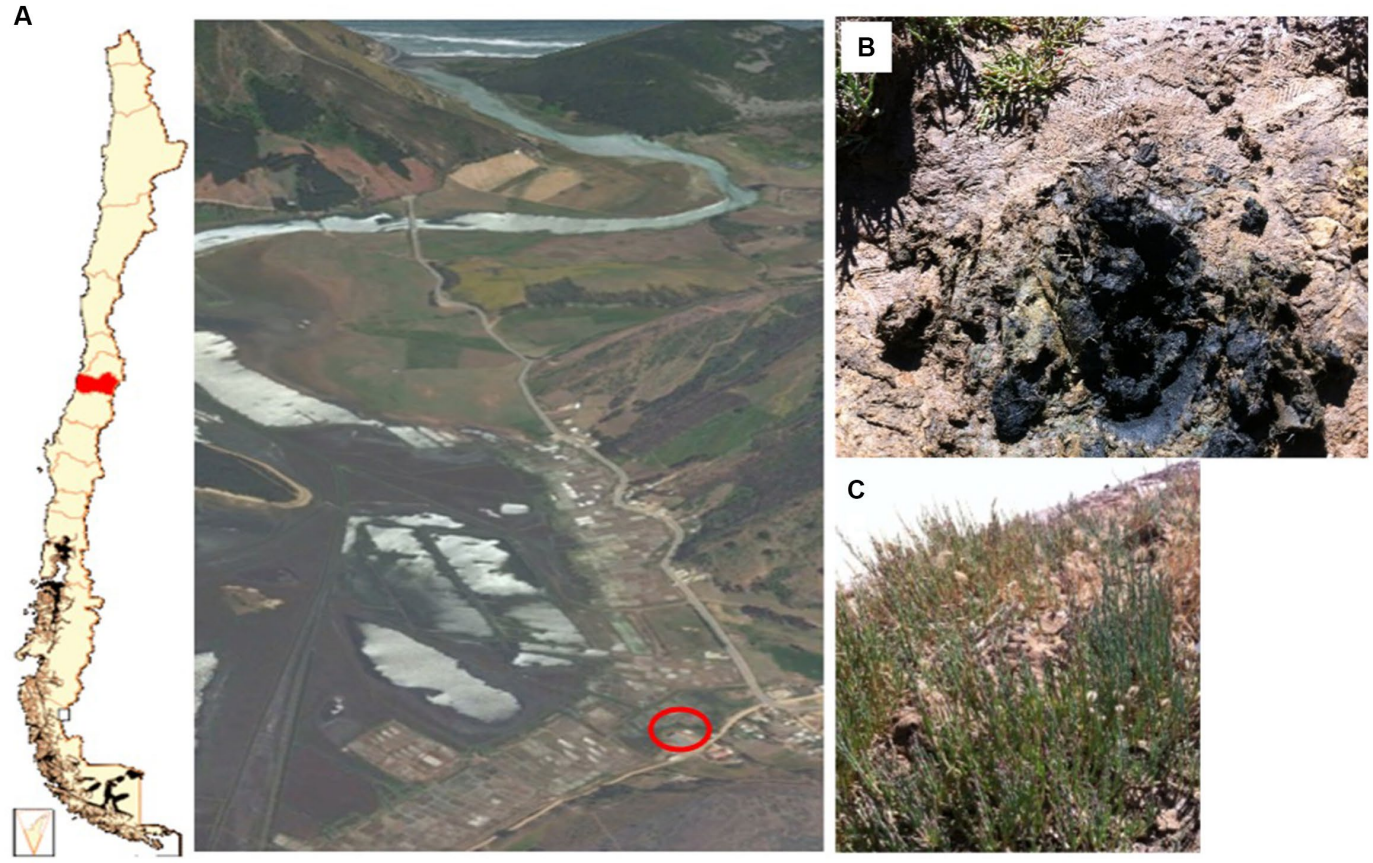
**(A)** Lo Valdivia solar salterns localization and an aerial view of Lo Valdivia estuary. **(B)** Specific sample point on Lo Valdivia solar saltern. **(C)** *Allenrolfea vaginata* plants found on the site. The red circle indicates the exact area of the sample site.

Soil samples were obtained in January 2013 from Lo Valdivia solar saltern, a sea-salt production site located in central Chile (34°41′50.16″S, 72°00′44.3″W), a place free of AC contamination from agro-industrial sources. Soil samples were collected at six points around the salt ponds (1–1.5 m from the pond edge), where *A. vaginata* was profusely present and thus defined as *A. vaginata* rhizosphere soil samples (Figure 1). Soil samples were collected to a maximum depth of 15 cm and stored in a plastic bag. The soil samples, free of root pieces and small stones, were mixed and homogenized and then passed through a 3 mm mesh to prepare the microcosms and perform chemical characterization. The latter was carried out in the Faculty of Agriculture and Forestry laboratory at the Pontificia Universidad Católica de Chile, following standard protocols (Robertson et al., 1999). A fresh, not sifted soil sample was used to isolate rhizosphere halophilic microorganisms.

Eight sets of nine microcosms each were set up with homogenized rhizosphere soil samples as follows: 100 mL beakers were filled with 10 g of soil, covered with self-sealing Parafilm M (Bemis, WI, United States), and incubated at room temperature (25°C ± 2°C). Three sampling times were established (1, 15, and 30 days of incubation). At each sampling time, three microcosms from each set were sacrificed, 2 g homogenized soil samples from each microcosm were used for DNA extraction, and the remaining for HPLC analysis. Each microcosm was supplemented with 1 mL of a solution containing 2,4-D, 4-HB, Phe, or Tyr at 5- or 20-mM final concentration. In addition, two control microcosms were used: one treatment without any AC addition and another where only 1 mL of sterilized distilled water was added instead of AC.

### Determination of AC concentration in soil samples

Five grams of each saline soil microcosm were mixed with 15 mL of methanol in a conical bottom tube of 50 mL and stirred for 24 h at 200 rpm. The mixture was then centrifuged at 10,860 g for 45 min to recover the supernatant. The supernatant solution was filtered sequentially through 0.45 μm and 0.22 μm pore size filters. To determine the corresponding AC content, the final solution was analyzed by HPLC. HPLC was performed at the Universidad de Chile soil science laboratory in an instrument connected with a Waters 1525 HPLC binary pump and a Waters 2996 diode array. Samples were isocratically eluted using an aqueous solution of phosphoric acid (3%) at 1.5 mL min^−1^ flow and a mobile phase of 60:40 (methanol/water), using a Kromasil column 100–3.5C18, dimension 4.6 × 150 mm. Absorbance was measured at 228, 254, 211, and 274 nm to detect 2,4-D, 4-HB, Phe, and Tyr, respectively. Data were managed, visualized, and statistically analyzed using RStudio software (RStudio Team, 2020). Statistical analysis of aligned-rank transform (Wobbrock et al., 2011) was applied to detect significant differences among compound and time factors. “*ARTool*” package was used to perform ANOVA of aligned rank-transformed data (Elkin et al., 2021), and the packages “*emmeans*” (Lenth et al., 2018) and “*multcomp*” (Hothorn et al., 2008) were used to perform pairwise contrasts.

### T-RFLP analysis

Molecular analysis of bacterial and archaeal communities was performed using metagenomic DNA isolated from 1 g of soil from each microcosm soil sample using the FastDNA SPIN Kit for Soil (MP Biomedicals, Santa Ana, California, United States) according to the manufacturer’s instructions. Amplification of *16S rRNA* gene was carried out by polymerase chain reaction (PCR) using the primer pairs 63F (6-FAM-5′-AGGCCTAACACATGCAAGTC-3′) and 1087R (5′-CTCGTTGCGGGACTTACCCC-3′) for bacteria domain (Singh et al., 2006), and 21F (6-FAM-5′-TTCCGGTTGATCCTGCCGGA-3′) and 1492R (5′-GGTTACCTTGTTACGACTT-3′) for archaea domain (Waddell et al., 2010). Both forward primers were labeled with the fluorophore 6-carboxy-fluorescein (6-FAM) at the 5′ terminal end. The reaction mixture for the PCR amplification included 5 μL 10× PCR buffer (200 mM Tris-HCl, pH 8.4, 500 mM KCl), 3.5 μL of 25 mM MgCl_2_, 1 μL each of primer 63F/1087R (0.2 μM) for bacterial sequences and 21F/1492R (0.2 μM) for archaeal sequences, 1 μL of dNTP (0.2 mM), 1 μL of bovine serum albumin (0.5 mg mL^−1^), 0.4 μL of 1 U Taq DNA polymerase, 2 μL of environmental DNA, in a total reaction volume of 50 μL. Amplification was performed using the Applied Biosystems 2720 Thermocycler (Applied Biosystems, Foster City, CA, United States). Reactions were held at 95°C for 10 min to allow the DNA to denature, followed by 25 cycles at 95°C for 45 s, 56°C for 1 min, and 72°C for 2 min, followed by a final extension at 72°C for 7 min. PCR products were visualized by electrophoresis on a 1% (w/v) agarose gel using GelRed as the DNA intercalating agent. The PCR products were subjected to digestion with the restriction enzymes *Alu*I and *Msp*I at 37°C for three h, followed by 20 min inactivation at 80°C. After digestion, products were purified with 0.1 vol of 3 M sodium acetate and 2.5 vol 100% ethanol and centrifuged at 26,290 g for 30 min at 4°C. After washing with 100 μL of 70% ethanol and centrifuging, DNA was eluted using 10 μL of ultra-pure water. Restriction fragment size analysis was performed through capillary electrophoresis (Macrogen Inc., Korea). Data from profiles of the restriction fragments were read in Peak Scanner version 1.0 Software (Applied Biosystems) using the standard marker 1200 LIZ and exported as a text format archive. Exported data were read and managed using RStudio software (RStudio Team, 2020). Only restriction fragments between 50 and 500 bp were considered for analysis. Fragments comprising less than 1% relative abundance were excluded from the study. The data were standardized by calculating the area of each peak as a percentage of the total area (Osborne et al., 2006). Using the R-package vegan (Oksanen et al., 2020), data were square root transformed, and the Bray–Curtis distance was calculated. The similarity matrix was used to represent the data with an NMDS (Clarke, 1993), and treatment clusters were highlighted using the ordiellipse function. Each compound/concentration cluster was highlighted using the ordispider function of the R package vegan (version 2.5–7) (Oksanen et al., 2020). The ANOSIM test, equivalent to a non-parametric ANOVA, was used to test similarity distances. Ecological diversity was also calculated with the Shannon–Weaver index (*H*′ = *S* pi * ln (pi)) and richness index (*S*) as the total number of OTUs found (Shannon, 1948; Blackwood et al., 2007).

### Enrichment, isolation, and identification of AC removing halophilic microorganisms

To isolate halophilic microorganisms capable of AC removal, 2 g of saline soil were mixed in a 500 mL flask with 100 mL of the modified HM liquid medium (Ventosa et al., 1982). This medium (HTM) contained 117 g of NaCl, 29.7 g of MgSO_4_, 20.7 g MgCl_2_, 3 g KCl, 0.43 g CaCl_2_, 0.39 g NaBr, and 0.1 g HNaCO_3_ with 10 mL of 10× phosphate buffer and 10 mL of the micronutrient mix to make 1 L, adjusted to pH = 7.2. To isolate halophiles, HTM medium salt concentration was adjusted to 4.5% salinity by diluting the medium with distilled water. 2,4-D, 4-HB, Phe, or Tyr were added at a 5 mM concentration, and flasks were incubated for 1 month at 25°C. After that, aliquots of 100 μL of a 1:1000 cultures dilution were plated on an HTM medium containing 1.5% agar and 1 mM of the corresponding AC. The agar plates were incubated for 1 month at room temperature. Microorganisms showing visible growth and potentially degrading AC were selected and transferred to a new selective solid medium HTM (4.5% salinity, equivalent to EC_susp_ = 43.8, Table 1, based on the equation EC = 9.7381*Salinity + 0.9771; *R*^2^ = 0.9987) containing the respective compound.

**Table 1:** Chemical characterization of *Allenrolfea vaginata* rhizosphere soil from Lo Valdivia solar saltern.

Parameter	Unit	Value
pH (H_2_O)	—	7.15
EC susp	mS cm^−1^	43.8
EC ext	mS cm^−1^	201.0
O.M.	%	2.09
*B*	mg Kg^−1^	17.75
(SO_4_)^2−^	meq L^−1^	340.0
Cl^−^	meq L^−1^	1637.0
(HCO_3_)^−^	meq L^−1^	1.7
Na^+^	meq L^−1^	983
Ca^2+^	meq L^−1^	44
Mg^2+^	meq L^−1^	662
K^+^	meq L^−1^	33
SAR		52.3

SO_4_^2−^, Cl^−^, (HCO_3_)^−^ and Na^+^, Ca^2+^, Mg^2+^, K^+^ values correspond the concentrations of soluble anions and cations, respectively. *B* value corresponds to available boron in soil. EC susp, electrical conductivity of the suspension, EC ext, electric conductivity of the extract, O.M., organic matter; SAR, sodium absorption ratio.

One isolated colony was used as inoculum for liquid culture, using the same diluted HTM medium and the respective compound where each colony was originally cultured. An aliquot of 50 μL was taken when turbidity was observed and plated in a solid medium containing 500 mL of HTM medium plus 5 g tryptone, 2.5% yeast extract, and 1.5% agar. Forty-one isolated colonies were selected for taxonomical identification based on 16S ribosomal gene sequencing. A colony was taken and resuspended in 800 μL of Chelex100 (BioRad, Hercules, CA, United States) for DNA extraction. The mixture was incubated at 80°C for 30 min and immediately chilled on ice for 5 min. Each mix was centrifuged at 26,290 g for 15 min, and the genomic DNA supernatant was recovered. Genomic DNA extracts were amplified by the above PCR program for T-RFLP analysis, using no fluorescent labeled primer pairs 63F and 1087R or 21F and 1492R. The PCR products were sent to sequencing (Macrogen Inc., Korea). The sequences were analyzed using BLAST (NCBI database GenBank). The *16S rRNA* gene sequences have been deposited in the GenBank database under accession numbers MZ322696- MZ322737.

To explore the ability of the isolates to grow on the four AC tested, HTM saline medium was prepared at 4.5% salinity, and ACs were added at 0.5, 1.0 or 2.0 mM final concentration. Non-inoculated, sterile cultures were used as controls. To determine the growth rates, cell concentrations were monitored at 600 nm (UV–VIS Spectrophotometer, Shimadzu, Kyoto, Japan) after 1, 3, 7, 14, 30, and 60 days of incubation at 200 rpm and 25°C. To quantify AC removal rates, 1 mL samples of each culture and controls were filtered with a 0.02 μm pore size filter and monitored by UV-spectrophotometry. Absorbance was measured at 228, 254, 211, and 274 nm to quantify 2,4-D 4-HB, Phe, and Tyr concentration, respectively, and removal percentages were estimated using the formula (([control] − [sample])/[control])*100. GraphPad Prism version 6.0.0 for Windows (GraphPad Software, San Diego, California United States, www.graphpad.com) was used to visualize data.

### Plant growth tests

Five halophilic microorganisms were selected to evaluate their ability to protect *A. thaliana* plants (Col-0) under salinity conditions and AC chemical stress. *A. thaliana* seeds were obtained from the Arabidopsis Biological Resource Center (ABRC, Columbus, OH, United States). The selected isolates were *Halomonas* sp. LV-8T, *Alkalihalabacillus* sp. LV-39H, *Thalassobacillus* sp. LV-41P, and *Marinobacter* sp. LV-48T, representing the genera with the most significant number of isolates. The archaeal isolate *Haladaptatus* sp. LV-51T was also included as the only isolate belonging to this kingdom. A plant growth-promoting bacteria, *Paraburkholderia phytofirmans* PsJN (Poupin et al., 2013), was included as a positive control by its ability to promote the growth of *A. thaliana* under saline stress (Pinedo et al., 2015). Halophile isolates were routinely grown in (100 mM NaCl/10 mM CaCl_2_) saline R2A liquid medium, and *P. phytofirmans* PsJN was grown in the same medium without salt added. Cell suspensions from each inoculum were then collected and adjusted to approximately 10^8^ colony-forming units per milliliter (CFU/mL), as determined by plate counting. Seeds were surface sterilized with 50% sodium hypochlorite 100% commercial laundry bleach containing 0.1% Tween 20, rinsed three times with sterile water, and kept at 4°C for 5 days to synchronize germination. Square Petri dishes were prepared with half-strength (Murashige and Skoog 1962) medium (MS½), 0.8% agar. To prepare the inoculated plates, the initial inoculum (10^8^ CFU/mL) was homogenously diluted in MS½ 0.8% agar just before gelling to reach a final concentration of 10^4^ CFU/mL of medium. This final inoculum size has been proven optimal for exploring plant growth promotion features (Poupin et al., 2013), but does not represent true environmental relative abundances. Sterilized and synchronized seeds were sown in the Petri dishes with MS½ medium inoculated or not with one strain. Plates were placed vertically in a growth chamber at 22°C with a photoperiod of 12/12 h (light/dark) for 7 days after transplant to specific conditions according to experimental design.

After 7 days of sowing (7 DAS), 16 *A. thaliana* seedlings per treatment were transplanted to a new square Petri dish containing the same volume and amount of agar without inoculation. Two treatments per strain were tested, one containing MS½ medium and a concentration of 100 mM NaCl plus 10 mM CaCl_2_ and another in which only MS½ medium was used. Three non-inoculated treatments (WO_MO treatments) were also run: one in the presence of AC, the other under saline conditions, and a third one combining both AC and salty conditions. Two plates per treatment with 15 seedlings each were incubated in a growth chamber under the same conditions described above for 14 days. At the end of the incubation period, the plates were photographed using a scanner (Epson, Perfection V600 Photo, Nagano, Japan), the root length was measured, and the number of leaves was counted with the naked eye. Subsequently, by analyzing the images taken on day 0 (7 DAS) and day 14 (21 DAS), plant growth was calculated as the difference in the rosette area between 0 and 14 days of incubation.

The effect of AC on the germination rate was tested by sterilizing and synchronizing 500 seeds and subjecting a fraction of these to different AC concentrations using the same medium and conditions described above. A concentration of 0.1 mM of phenol and 2,4-D, 0.5 mM of 4-HB, and Tyr were determined to be toxic to *A. thaliana*.

### Image analysis

The rosette area was image analyzed using Adobe Photoshop Cs3 software. This software allows measurement of the number of pixels corresponding to the area of the Petri dish used (12 × 12 cm). Since all the images were captured at the same resolution and height, this parameter was used to estimate the rosette area, converting the number of pixels into cm^2^. Rosette area data was obtained from images taken on days 1 and 14.

## Results

This work aimed to improve the knowledge of the role of microbial communities associated with halophyte plants, focusing on the effects of ACs. Effects on microbial community structure were evaluated using saline soil microcosms exposed to these ACs and terminal restriction fragment length polymorphism (T-RFLP) fingerprinting analysis. AC removal was determined by high-performance liquid chromatography (HPLC). Conventional culture enrichment and isolation procedures were also used to obtain and preliminary characterize bacteria able to grow on these ACs. *In vitro* experiments explored the individual capacities of halophiles to promote the growth of *Arabidopsis thaliana* under saline stress conditions.

### Chemical characterization of saline soil

*Allenrolfea vaginata* rhizosphere soil possessed a high content of soluble anions and cations (Table 1), leading to 4.5% salinity. The most conspicuous anions presented were chlorides and sulfates, and cations such as Na^+^ and Mg^2+^ were the most abundant in this soil. At the same time, the organic matter was found in the normal range for agricultural soils (Table 1). In addition, the high electrical conductivity of the extract and suspension at neutral pH indicated that this soil might be classified as saline-sodic (Table 1).

### Removal of AC in saline soil microcosms

**Figure 2:**
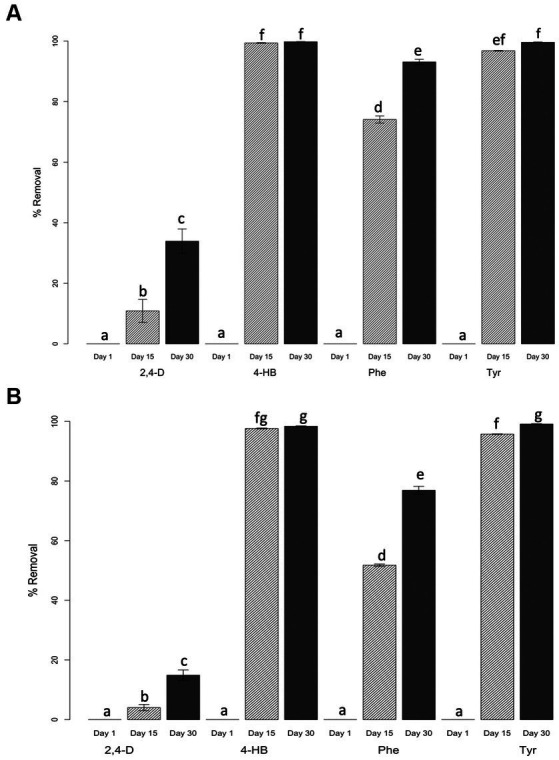
Percent of aromatic compound removal in saline soil microcosms. Initial concentrations of xenobiotic compounds were 5 mM **(A)** and 20 mM **(B)**. Standard deviations represented by the error bars were calculated using triplicate biological samples. Letters on top of each column represent statistical differences of post-hoc contrasts art-c test analysis using the Tukey method.

Rhizosphere saline soil microcosms were spiked with five and 20 mM of 2,4-D, 4-HB, Phe, or Tyr and monitored on days 1, 15, and 30 using HPLC analysis to quantify soil concentration of each compound and calculate removal percentages (Figure 2). At day one no removal of these compounds was observed, while at day 15, almost all 4-HB and Tyr were removed, at both concentrations. Full removal levels at 5 mM and 20 mM were observed after 30 days for 4-HB (99.8 ± 0.1% and 99.6 ± 0.2%, respectively) and Tyr (98.4 ± 0.3% and 99.1 ± 0.3%, respectively). 2,4-D and Phe removal levels increased in time but were lower than the other two compounds. 2,4-D was the less-removed compound as maximum removal levels were 33.9 ± 7.0% and 14.9 ± 1.1% at 5 and 20 mM, respectively. Phe was significantly removed at 5 mM (93.1 ± 1.5%), but removal levels decreased at 20 mM (76.9 ± 2.3%). Nonparametric statistical analysis of variance of aligned rank transformed data (Wobbrock et al., 2011) confirmed that significant differences in removal were due to time and compound factors interaction at both concentrations 5 mM (*F*_6, 24.0_ = 114.726, *p* = 2.1852E−11) and 20 mM (*F*_6, 24.0_ = 87.062, *p* = 4.3228E−15). A post-hoc pairwise Tukey test showed that significant differences were associated with the non-removal of any compound at day one and removal differences in time of each compound under both compound concentration treatments (Figure 2).

### Alpha diversity analysis of rhizosphere saline soil bacterial and archaeal community

To assess the effects of AC on bacterial and archaeal communities, T-RFLP profiles of saline soil microcosms were used to calculate ecological indexes of diversity (*H*) and richness (*S*). *S* and *H* values for bacterial communities were consistently higher than archaeal communities (Table 2). Archaeal indexes showed almost no variation in time, with differences present only in one or two operational taxonomic units (OTU). The two control conditions not amended with AC displayed higher levels of S and H in archaeal communities than treated microcosms in archaeal communities. Compared with these two controls, bacterial communities were rapidly and strongly affected by the exposure to AC, increasing the number of OTU detected on day 1 (Table 2). When Phe was added, *S* decreased after 15 and 30 days of incubation. A similar pattern was observed with the *H* index (Table 2). 2,4-D effects on community indexes highly depended on the compound concentration (Table 2). A noticeable increase of *S* and *H* was observed at day 15 with 20 mM 2,4-D. Tyr and 4-HB treatments produced similar changes in community index changes.

**Table 2:** Richness (*S*) and Shannon diversity (*H*) indexes based on terminal restriction fragment length polymorphism profiles of microbial communities from saline soil microcosms exposed to 5 mM or 20 mM aromatic compounds.

Index	Richness (*S*)			Diversity (*H*)		
Time	Day 1	Day 15	Day 30	Day 1	Day 15	Day 30
**Bacteria 5 mM**						
2,4-D	22.3 (0.58)	21	18	2.82 (0.02)	2.74 (0.03)	2.56 (>0.01)
4-HB	21	22	22	2.78 (0.03)	2.89 (>0.01)	2.90 (0.03)
Phe	22	12	13	2.88 (0.03)	1.94 (0.04)	2.20 (0.02)
Tyr	21	19	20	2.67 (0.09)	2.68 (>0.01)	2.33 (0.15)
Water	14	14	14	2.37 (>0.01)	2.35 (0.15)	2.40 (0.07)
Not-treated	14	14	14	2.37 (0.08)	2.18 (0.06)	2.45 (>0.01)
**Archaea 5 mM**						
2,4-D	3	3	4	0.65 (0.06)	0.62 (>0.01)	1.156 (0.03)
4-HB	3	3	4	0.89 (>0.01)	0.88 (>0.01)	0.8 (0.01)
Phe	3	3	4	0.72 (0.02)	0.82 (0.02)	0.95 (0.01)
Tyr	5	5	5	1.41 (>0.01)	1.39 (>0.01)	1.42 (>0.01)
Water	6	6	6	1.00 (0.02)	1.26 (>0.01)	1.33 (0.04)
Not-treated	6	6	6	1.25 (0.08)	1.34 (0.03)	1.32 (0.04)
**Bacteria 20 mM**						
2,4-D	18	25	18	2.46 (0.03)	2.93 (0.02)	2.57 (>0.01)
4-HB	21	20	23	2.77 (0.05)	2.64 (0.02)	2.73 (0.08)
Phe	18	15	14	2.57 (0.03)	2.00 (0.05)	2.08 (0.1)
Tyr	22	18	20	2.65 (0.04)	2.51 (0.06)	2.72 (0.03)
Water	14	14	14	2.37 (>0.01)	2.35 (0.15)	2.40 (0.07)
Not-treated	14	14	14	2.37 (0.08)	2.18 (0.06)	2.45 (>0.01)
**Archaea 20 mM**						
2,4-D	3	4	4	0.64 (0.03)	0.95 (0.02)	0.80 (>0.01)
4-HB	3	4	4	0.79 (>0.01)	0.86 (0.02)	0.90 (0.02)
Phe	3	3	4	0.69 (>0.01)	0.81 (>0.01)	0.95 (0.01)
Tyr	5	5	5	1.41 (>0.01)	1.39 (>0.01)	1.42 (>0.01)
Water	6	6	6	1.00 (0.02)	1.26 (>0.01)	1.33 (0.04)
Not-treated	6	6	6	1.25 (0.08)	1.34 (0.03)	1.32 (0.04)

Values represent the average of three samples; standard deviations not equal to zero are shown in parentheses. Values from microcosm without treatment (not treated) or without aromatic compound (water), are also shown.

### Beta diversity analysis of rhizosphere saline soil bacterial and archaeal communities

**Figure 3:**
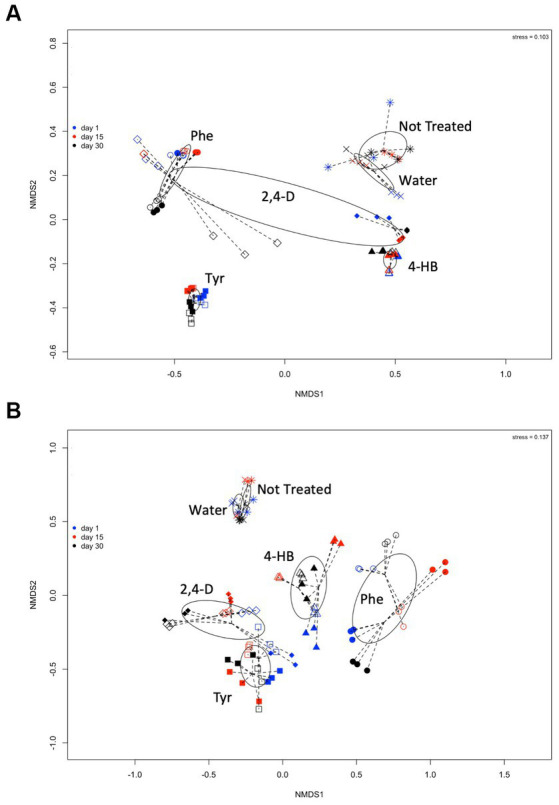
Comparisons of **(A)** archaeal and **(B)** bacterial community composition from saline soil microcosm experiments assessed by non-metric multidimensional scaling of *Msp*I-terminal restriction fragment length polymorphisms (T-RFLP) of the *16S rRNA* gene analysis. Each symbol corresponds to a single T-RFLP profile representing one of the three replicates from a single community structure at day 1 (blue symbols), day 15 (red symbols), and day 30 (black symbols), exposed to 5 mM (open diamonds) or 20 mM (solid diamonds) 2,4-D; to 5 mM (open triangles) or 20 mM (solid triangles) 4-HB; to 5 mM (open circles) or 20 mM (solid circles) Phe, and 5 mM (open squares) or 20 mM (solid squares) Tyr. T-RFLP profiles from microcosm controls without treatment (*) or without aromatic compound, water-only treatment (x), are also shown. Ellipses and lines were drawn using ordiellipse and ordispider functions from the vegan package.

The effects of these ACs on the changes in the structure of the saline soil rhizosphere bacterial and archaeal communities were further studied. Non-metric multidimensional scaling (NMDS) comparison of the *16S rRNA* gene T-RFLP profiles showed a clear grouping of samples according to treatment (Figures 3A,B). Phe and 2,4-D treatments presented higher dispersion in the NMDS ordering. Particularly noticeable, the 5 mM 2,4-D treatment showed a more substantial dispersion, mainly in archaeal communities (Figure 3A). The finding that the compound component is the more significant dissimilarity factor was confirmed by the analysis of similarity (ANOSIM), achieving a global *R* of 0.7433 (*p*-value = 0.001) for bacterial communities, and 0.7802 (*p*-value = 0.001) for archaeal communities. The *R* statistic parameter indicates the strength of the difference where 1 is the strongest and 0 is the weakest, representing significant differences between treated communities. T-RFLP profiles from non-treatment or water-only treatment were also clearly different (Figure 3). The results indicated that the increase of 2,4-D concentration had a more significant effect on archaeal community composition (Figure 3A open and solid diamonds) than on bacterial community (Figure 3B). This phenomenon of compositional divergence produced by different concentrations was not observed with the other compounds (Figure 3). On the other hand, there were no signs of resilience within the 30 days, as each compound generated compositional changes that, in general, accentuated through time (Figure 3, black symbols). The pairwise analysis showed that two controls (non-treatment & water-only) presented *R* values of 0.667 and 0.556 for archaea and bacteria, respectively (Table 3), which indicates that water addition had a low effect on microbial community structure changes. These two controls showed different results at 15 and 30 days, as *R* values were 1 and 0.481 in archaeal and 0.296 and 0 for bacterial communities, respectively (Table 3). This indicates that the bacterial community structural differences between no-treatment and water-only treatment decreased over time, while archaeal community structural differences peaked at day 15. Bacterial communities exposed to AC showed a fast change in composition after each compound was added (compare treatments and controls), and the amended compound mainly explained the observed differences.

**Table 3:** ANOSIM Pairwise *p*-values calculated using T-RFLP data.

Pairs	ALL		Day 1		Day 15		Day 30	
	Archaea	Bacteria	Archaea	Bacteria	Archaea	Bacteria	Archaea	Bacteria
Tyr_5 mM/Tyr_20 mM	−0.041	0.059	0.259	1	−0.333	1	0.852	1
Tyr_5 mM/Phe_5 mM	1	1	1	1	1	1	1	1
Tyr_5 mM/Phe_20 mM	0.976	1	1	1	1	1	1	1
Tyr_5 mM/4-HB_5 mM	1	0.623	1	1	1	1	1	1
Tyr_5 mM/4-HB_20 mM	1	0.38	1	1	1	1	1	1
Tyr_5 mM/2.4-D_5 mM	0.834	0.821	1	1	1	1	1	1
Tyr_5 mM/2.4-D_20 mM	1	0.692	1	1	1	1	1	1
Tyr_5 mM/Water	1	1	1	1	1	1	1	1
Tyr_5 mM/W.T.	1	1	1	1	1	1	1	1
Tyr_20 mM/Phe_5 mM	1	1	1	1	1	1	1	1
Tyr_20 mM/Phe_20 mM	0.972	1	1	1	1	1	1	1
Tyr_20 mM/4-HB_5 mM	1	0.767	1	1	1	1	1	1
Tyr_20 mM/4-HB_20 mM	1	0.579	1	1	1	1	1	1
Tyr_20 mM/2.4-D_5 mM	0.833	0.907	1	1	1	1	1	1
Tyr_20 mM/2.4-D_20 mM	1	0.83	1	1	1	1	1	1
Tyr_20 mM/Water	1	1	1	1	1	1	1	1
Tyr_20 mM/W.T.	1	1	1	1	1	1	1	1
Phe_5 mM/Phe_20 mM	0.025	0.076	0.778	1	1	1	0.556	1
Phe_5 mM/4-HB_5 mM	1	0.941	1	1	1	1	1	1
Phe_5 mM/4-HB_20 mM	1	0.953	1	1	1	1	1	1
Phe_5 mM/2.4-D_5 mM	0.309	1	1	1	1	1	1	1
Phe_5 mM/2.4-D_20 mM	1	0.976	1	1	1	1	1	1
Phe_5 mM/Water	1	1	1	1	1	1	1	1
Phe_5 mM/W.T.	1	1	1	1	1	1	1	1
Phe_20 mM/4-HB_5 mM	1	0.748	1	1	1	1	1	1
Phe_20 mM/4-HB_20 mM	1	0.814	1	1	1	1	1	1
Phe_20 mM/2.4-D_5 mM	0.211	0.991	1	1	1	1	1	1
Phe_20 mM/2.4-D_20 mM	1	0.93	1	1	1	1	1	1
Phe_20 mM/Water	1	1	1	1	1	1	1	1
Phe_20 mM/W.T.	1	1	1	1	1	1	1	1
4-HB_5 mM/4-HB_20 mM	0.304	0.097	1	1	1	1	0.704	1
4-HB_5 mM/2.4-D_5 mM	0.836	0.872	1	1	1	1	1	1
4-HB_5 mM/2.4-D_20 mM	0.49	0.795	1	1	1	1	1	1
4-HB_5 mM/Water	1	0.991	1	1	1	1	1	1
4-HB_5 mM/W.T.	1	0.991	1	1	1	1	1	1
4-HB_20 mM/2.4-D_5 mM	0.844	0.741	1	1	1	1	1	1
4-HB_20 mM/2.4-D_20 mM	0.528	0.619	1	1	1	1	1	1
4-HB_20 mM/Water	1	0.988	1	1	1	1	1	1
4-HB_20 mM/W.T.	1	0.986	1	1	1	1	1	1
2.4-D_5 mM/2.4-D_20 mM	0.823	−0.041	1	1	1	1	1	0.407
2.4-D_5 mM/Water	1	1	1	1	1	1	1	1
2.4-D_5 mM/W.T.	1	1	1	1	1	1	1	1
2.4-D_20 mM/Water	0.997	1	1	1	1	1	1	1
2.4-D_20 mM/W.T.	1	1	1	1	1	1	1	1
Water/W.T.	0.669	0.1	0.667	0.556	1	0.296	0.481	0

W.T., Without treatment.

### Removal of 2,4-D, 4-HB, Phe, and Tyr by *Allenrolfea vaginata* rhizosphere saline soil isolates

**Figure 4:**
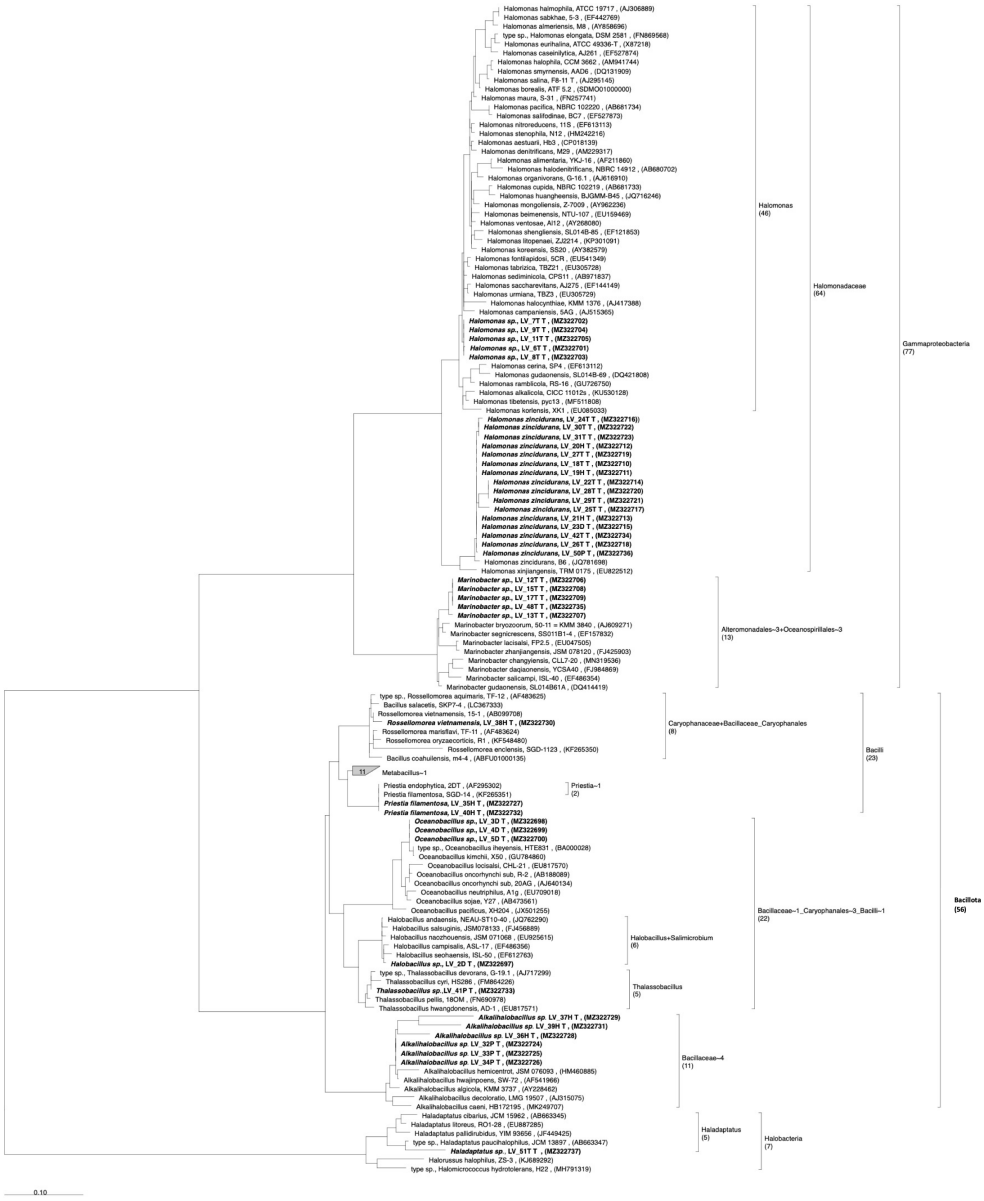
Phylogenetic analysis of the isolates from *Allenrolfea vaginata* rhizosphere soil, based on *16S rRNA* gene sequence, showing the taxonomic relationship between halophile bacteria and archaea isolates. The tree was constructed using the maximum likelihood method. Sequences for analysis were obtained from GenBank. Bootstrap values are shown next to the branches and were calculated as a percentage of 100 replicates. Isolated microorganism labels indicate the sample site (Lo Valdivia), the isolate number, and the final letter indicates the compound used for its isolation. Access numbers to the gene bank are shown in parentheses. The number of entries, new isolates, and reference sequences is indicated between parenthesis below the taxonomical group.

Microorganisms from the rhizospheric saline soil were tested for their ability to remove/degrade ACs. Forty-one isolates from *A. vaginata* rhizosphere saline soil were obtained using a solid saline medium containing one of the four AC as a sole carbon and energy source. *16S rRNA* sequencing analysis revealed that the 41 isolates corresponded to unique *16S rRNA* sequences having more than 1% of sequence dissimilarity between the closest sequence of the isolated microorganisms (Figure 4). From these 41 isolates, *Halomonas* genus was the most represented (21 isolates), of which 16 isolates corresponded to the *H. zincidurans* species and five isolates corresponded to possibly new *Halomonas* species, closely related to each other (Figure 4). The isolates included also six possible new species of *Alkalihalobacillus*, five likely new species of *Marinobacter*, three potential new species of *Oceanobacillus*, two isolates closely related to *Priestia filamentosa,* one isolate closely related to *Rossellomorea vietnamensis* and possible new species of *Halobacillus*, *Thalassobacillus* and *Haladaptatus* (one isolate each), the latter being the only archaeal isolate obtained (Figure 4). It should be noted that a cautious expression about possible new species was used as only part (about 1,000 bp) of the *16S rRNA* sequence was obtained. Among the 41 isolates, 22 were isolated using Tyr, nine using 4-HB, five using Phe, and five using 2,4-D as a sole carbon and energy source.

**Figure 5:**
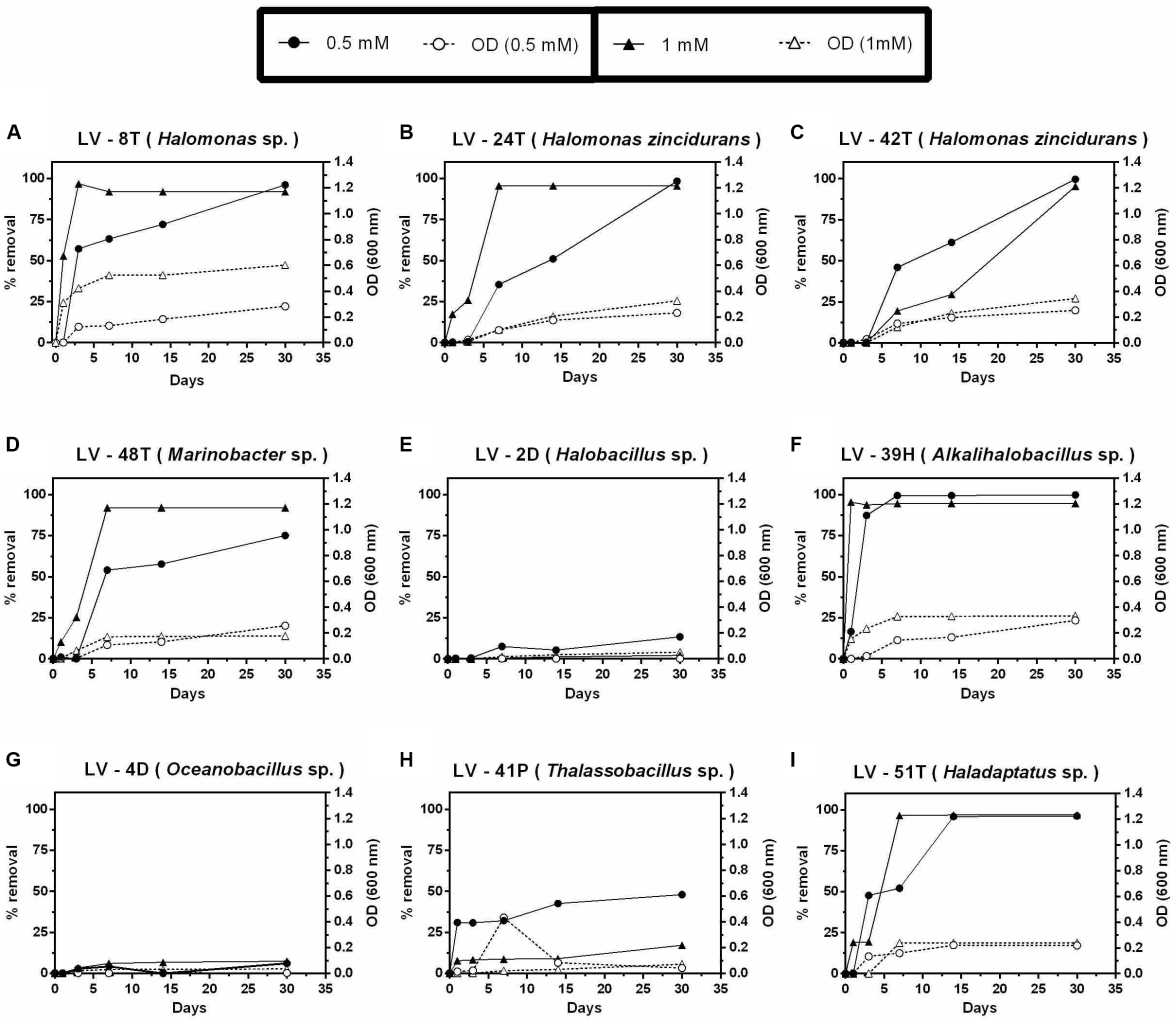
Growth and percent of 4-hydroxybenzoic acid (4-HB) removal by selected *Allenrolfea vaginata* rhizosphere saline soil isolates in incubations with two concentrations of 4-HB at 4.5% salinity. Growth (open symbols) was monitored at O.D._600 nm_, and 4-HB removal (closed symbols) was monitored at O.D._254 nm_. The more closely related species are indicated in parenthesis. Values correspond to triplicate averages. Standard deviations were lower than 10% and are not shown for clarity.

Nine isolates, including the single archaeal isolate, were selected for preliminary study AC removal versatility in liquid cultures, using two or three concentrations (0.5, 1.0, and 2.0 mM) of each of the four compounds at 4.5% salinity. Isolates of *Alkalihalobacillus*, *Marinobacter*, *Oceanobacillus, Halobacillus*, *Thalassobacillus*, *Haladaptatus,* and three *Halomonas* were selected, as they showed faster growth on solid agar plates. Despite an incubation of up to 60 days, none of these nine isolates could grow on 2,4-D at any concentration, including the *Halobacillus* sp. LV-2D and *Oceanobacillus* sp. LV-4D, which were initially isolated using 2,4-D as a growth substrate. Moreover, these two isolates were the only ones unable to grow and remove 4-HB (Figures 5E,G). *Thalassobacillus* sp. LV-41P showed low removal of 4-HB, presenting maximum removal levels of 48 and 17% at 0.5 and 1 mM after 30 days, respectively (Figure 5H). The other six isolates could almost completely remove 4-HB, although with different time courses (Figures 5A–D,F,I). *H. zincidurans* was the only isolate that performed better at 0.5 mM than 1 mM (Figure 5C).

**Figure 6:**
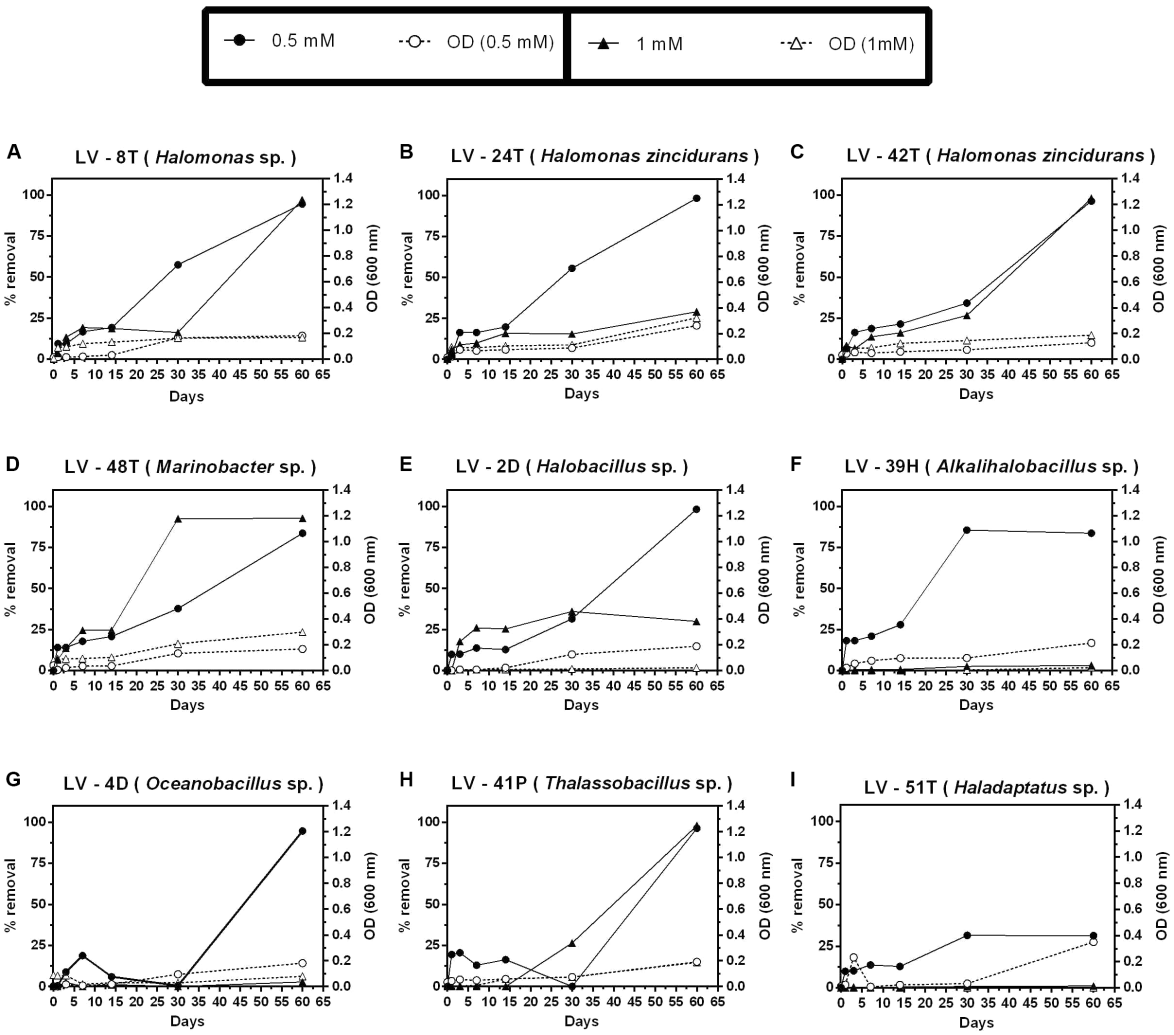
Growth and percent of phenol (Phe) removal by selected *Allenrolfea vaginata* saline rhizosphere isolates in incubations with two (Phe) concentrations at 4.5% salinity. Growth (open symbols) was monitored at O.D._600 nm_, and (Phe) removal (closed symbols) was monitored at O.D._211 nm_. The more closely related species are indicated in parenthesis. Values correspond to triplicate averages. Standard deviations were lower than 10% and are not shown for clarity.

In contrast to 4-HB, Phe removal was slower (Figure 6), as after 30 days, only *Marinobacter* sp. LV-48T attained significant removal of 0.5 mM Phe (Figure 6D), whereas complete removal was only observed after 60 days (Figures 6A–H), except for the archaeal isolate *Haladaptatus* sp. LV-51T (Figure 6I). Significant removal of 1 mM Phe was only detected in two *Halomonas* isolates (LV-8T and LV-42T) and both *Marinobacter* sp. LV-48T and *Thalassobacillus* sp. LV-41P (Figures 6A,C,D,H). As judged by O.D. 600 nm, biomass levels were lower than those observed with 4-HB.

**Figure 7:**
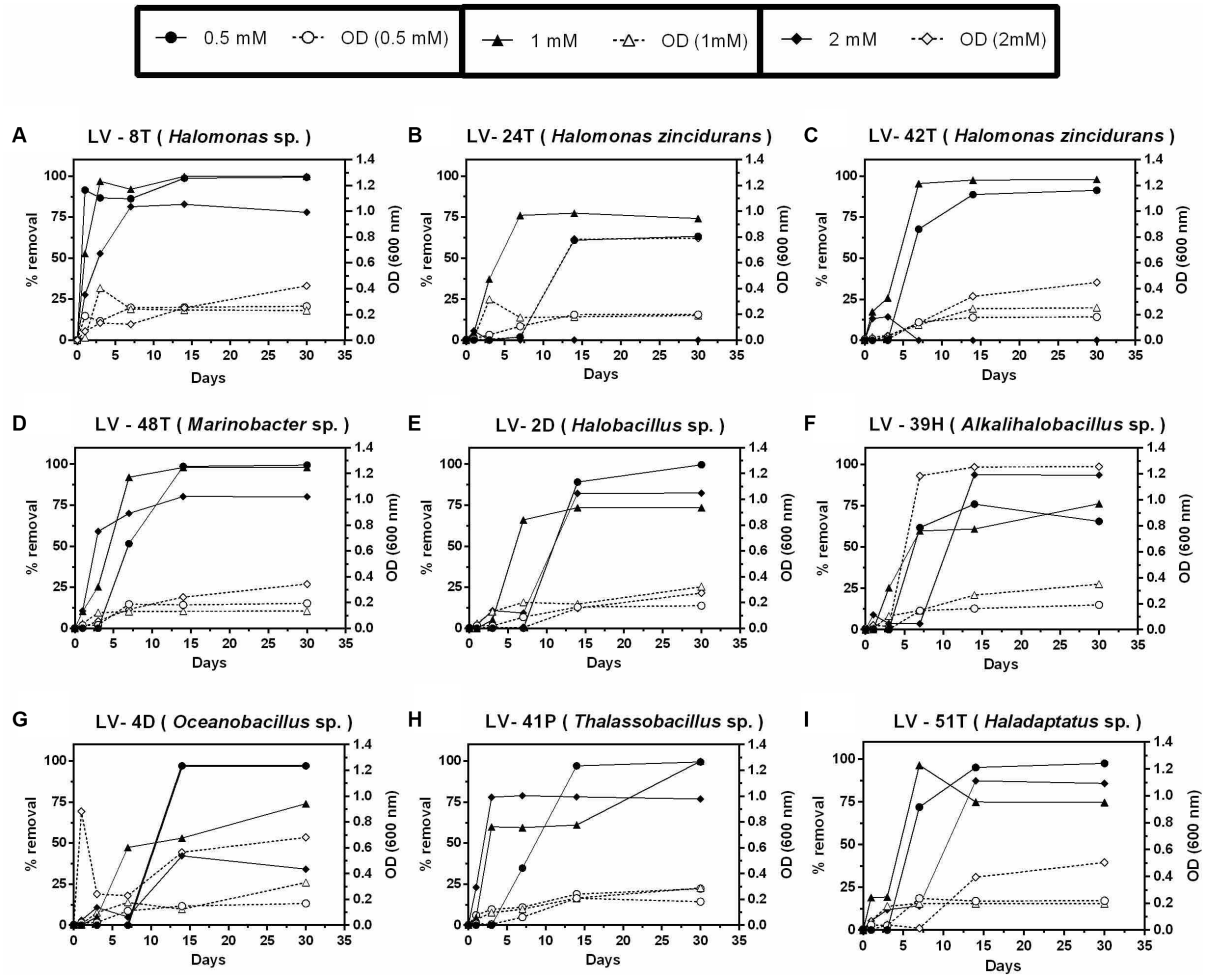
Growth and percent of tyrosine (Tyr) removal by selected *Allenrolfea vaginata* rhizosphere soil isolates in incubations with three Tyr concentrations at 4.5% salinity. Growth (open symbols) was monitored at O.D._600 nm_, and Tyr removal (closed symbols) was monitored at O.D._274 nm_. The more closely related species are indicated. Values correspond to triplicate averages. Standard deviations were lower than 10% and are not shown for clarity.

Tyr was the only compound that allowed growth and removal at 2 mM after 30 days (Figures 7A,D–F,H,I), except for isolates *H. zincidurans* LV-24T, LV-42T (Figures 7B,C), and *Oceanobacillus* sp. LV-4D (Figure 7G). Biomass levels, as judged by O.D._600 nm_, were similar to those observed with 4-HB.

### *Arabidopsis thaliana* plants growth promotion and protection by halophiles under saline stress

**Figure 8:**
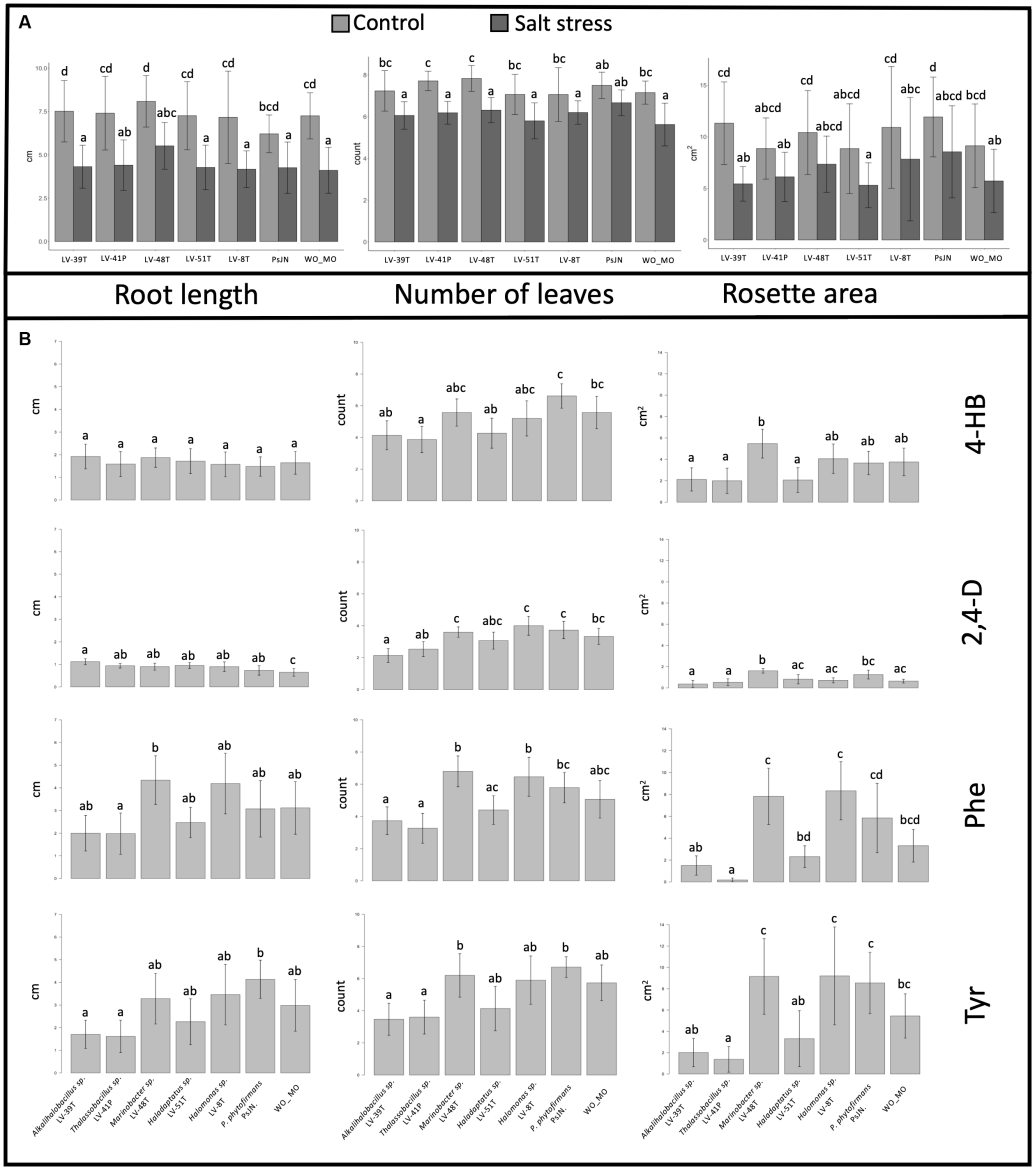
**(A)** Effects of selected halophilic microorganisms on *Arabidopsis thaliana* growth *in vitro*. Data were collected 14 days after transplantation. Bar graphics representation of root length (left panel), the number of leaves (central panel), and rosette area (right panel) of plants treated under saline stress and control conditions. Standard deviations represented by the error bars were calculated using 10 to 15 biological replicate plants. Letters on top of each column represent statistical differences of post-hoc contrasts art-c test analysis using the Tukey method. **(B)** Effects of selected halophilic microorganisms on *A. thaliana* growth *in vitro* under saline stress conditions and the presence or absence of aromatic compounds. Bar graphics representation of root length (left panel), the number of leaves (central panel), and rosette area (right panel) of *A thaliana* plants under saline conditions (100 mM salinity). Standard deviations represented by the error bars were calculated using ten to 15 biological replicate plants. Letters on top of each column represent statistical differences of post-hoc contrasts art-c test analysis using the Tukey method.

To address plant growth promotion and protection from saline stress by *A. vaginata* rhizosphere microorganisms, the interactions of *A. thaliana* Col-0 and five selected isolates or *P. phytofirmans* PsJN, a plant growth promotion rhizobacteria as positive PGPR control, were tested. At 7 DAS, plants were transferred to MS½ medium without salt (control) or with salt (100 mM NaCl/10 mM CaCl_2_). Plants were photographed 14 days after the transplant, and root length, number of leaves, and rosette area were measured. The rosette area values showed more significant variations, reflecting a two-dimensional parameter. In all the parameters measured, plants in control conditions (without salt) exhibited significant differences from those under saline stress (Figure 8A), represented by a greater root length (*F*_1, 6.205_ = 181.138, *p* < 2.22E−16), number of leaves (*F*_1, 6.205_ = 174.138, *p* < 2.22E−16) and rosette area (*F*_1, 6.203_ = 59.2656; *p* = 5.9191E−13). Under non-saline conditions, *Alkalihalobacillus* sp. LV-39H, and *Marinobacter* sp. LV-48T enhanced root length growth compared to the control PsJN or WO_MO (Figure 8A, left panel). No significant differences were found in the other three isolates relative to the WO_MO control. Curiously, strain PsJN did not promote root growth relative to the WO_MO control. Under saline stress conditions, the halophile strains LV-48T and LV-41P showed a clear root growth-enhancing effect (Figure 8A, left panel).

Under non-saline conditions, *Thalassobacillus* sp. LV-41P and *Marinobacter* sp. LV-48T showed promotion in the number of leaves compared to PsJN (Figure 8A, central panel). Still, the rest of the halophiles isolated did not present significant differences from the WO_MO control (Figure 8A, central panel). In contrast, only PsJN increased the number of leaves under saline stress with respect to WO_MO control (Figure 8A, central panel). Some halophiles also increased the rosette area under non-saline conditions, as shown for *Alkalihalobacillus* sp. LV-39H, *Marinobacter* sp. LV-48T, and *Halomonas* sp. LV-8T, compared to the WO_MO control (Figure 8A, right panel). Under this treatment, strain PsJN showed a more significant difference, promoting more growth than the halophiles (Figure 8A, right panel).

### *Arabidopsis thaliana* plants growth promotion and protection under saline stress by halophiles in the presence of aromatic compounds

To study growth promotion produced by these five selected halophiles in *A. thaliana*, under saline conditions and in the presence of AC, plants were exposed *in vitro* to salt stress, and in the presence of single AC. *A. thaliana* seeds were sown in MS½ medium with or without inoculation of selected halophiles, or *P. phytofirmans* strain PsJN used as a PGPR strain control. At 7 DAS, plants were transferred to MS½ medium containing salt (100 mM NaCl/10 mM CaCl_2_). Plants were photographed 14 days after the transplant, and root length, number of leaves, and rosette area were measured.

Regarding the effect of 4-HB on the growth of *A. thaliana* (Figure 8B), only two of the three plant growth parameters studied showed significant differences between treatments: the number of leaves (*F*_1, 6.94_ = 4.9788; *p* = 1.815E−4) and rosette area (*F*_1, 6.98_ = 4.2686; *p* = 7.304E−4). Pairwise comparisons through the Tukey test showed that the number of leaves was significantly greater when PsJN was inoculated than treatments inoculated with *Alkalihalobacillus* sp. LV-39H, *Marinobacter* sp. LV-48T, and *Halomonas* sp. LV-8T. However, none of these strains, including PsJN, showed significant differences in control treatment without microorganisms (WO_MO). However, it was observed that *Marinobacter* sp. LV-48T positively affected the rosette area (Figure 8B). Although there were other significant differences between treatments, they are inconclusive to ensure a positive or negative effect of microorganisms, as they do not differ significantly from the non-inoculated, negative control.

The 2,4-D presence provoked detrimental effects on *A. thaliana.* The statistical results showed differences between treatment root length (*F*_1, 6.93_ = 2.926, *p* = 1.1167E−2), number of leaves (*F*_1, 6.93_ = 7.297, *p* = 2.09E−6), and rosette area (*F*_1, 6.94_ = 8.1197; *p* = 4.448E−7). However, pairwise analysis showed differences between the control WO_MO and all treatments, indicating that halophile and PsJN strains may protect *A. thaliana* exposed to 2,4-D under salt stress. Post-hoc contrast analysis showed a significant increase in rosette area in the presence of *Marinobacter* sp. LV-48T, compared to the control WO_MO (Figure 8B).

Statistical analysis applied to the data related to the presence of Phe and salt stress on the growth of *A. thaliana* showed significant differences associated with root length (*F*_1, 6.95_ = 2.7981, *p* = 1.499E−2), number of leaves (*F*_1, 6.95_ = 1.1217, *p* = 4.296E−7) and rosette area (*F*_1, 6.96_ = 12.856; *p* = 1.3E−10). A growth promotion effect on root length under salt and Phe stress was observed in treatments with *Marinobacter* sp. LV-48T and *Thalassobacillus* sp. LV-41P compared to the control WO_MO (Figure 8B). However, strain LV-41P negatively affected the root length of *A. thaliana*. On the other hand, the number of leaves decreased in the presence of *Alkalihalobacillus* sp. LV-39H or *Thalassobacillus* sp. LV-41P compared to the results obtained in treatments where *Marinobacter* sp. LV-48T, and *Halomonas* sp. LV-8T were present. However, these results were not significantly different from those of the control WO_MO, indicating that both promotion and detrimental effects by those microorganisms are mild effects on plants (Figure 8B). Similar results were obtained when *P. phytofirmans* PsJN was present, which showed a promoter/protective effect but to a lesser extent than the halophiles *Halomonas* sp. LV-8T, and *Marinobacter* sp. LV-48T. Similarly, the rosette area decreased in the presence of *Thalassobacillus* sp. LV-41P compared to control WO_MO, in *A. thaliana* exposed to salt stress conditions and Phe (Figure 8B).

The presence of Tyr under saline stress showed significant differences in root length (*F*_1, 6.92_ = 3.2172, *p* = 6.4991E−3) between plants inoculated with strain PsJN compared to those inoculated with *Alkalihalobacillus* sp. LV-39H, and *Thalassobacillus* sp. LV-41P. Nevertheless, these results do not allow us to conclude whether these halophiles positively or negatively affect root length in *A. thaliana* because there were no significant differences compared to the control WO_MO (Figure 8B). Moreover, statistical analysis showed differences in leaf numbers of *A. thaliana* between treatments inoculated with *Marinobacter* sp. LV-48T and PsJN compared to those inoculated with *Alkalihalobacillus* sp. LV-39H and *Thalassobacillus* sp. LV-41P. Differences were observed between the control WO_MO and any of the five halophiles studied. Finally, rosette area significantly decreased in plants inoculated with *Thalassobacillus* sp. LV-41P, compared to the control WO_MO (Figure 8B).

## Discussion

This work studied the rhizosphere microbial community of the halophyte *A. vaginata* thriving in soil from Lo Valdivia solar salterns, the most austral salterns in the world. This study focused on AC’s effects on microbial community structure, its depurative potential, a preliminary characterization of microbial isolates growing on such compounds, and an assessment of the effects of these isolates on plant growth promotion and protection to saline stress, using *A. thaliana* as a model.

The rhizosphere soil used had high conductivity, exceeding four times the conductivities presented in coastal saline soils, which would likely reduce microbial degradation of organic compounds, as high salinity produces water stress in microbial communities (Li et al., 2019). Moreover, salinity decreases the amount of available oxygen, adversely affecting the activity of enzymes such as oxygenases involved in the degradation of organic compounds (Pérez-Pantoja et al., 2010a,b; Campbell and Kirchman, 2013; Guo et al., 2016). In addition, it has been proposed that over 1% of organic matter is required to ensure microbial activity associated with the degradation of organic compounds (Witzgall et al., 2021). The rhizosphere saline soil used in this work had a 2.1% organic matter content, thus positively influencing the high removal levels detected in microcosm experiments.

Salinity is a significant factor influencing the structure of microbial communities, with differential degrees of resilience depending on the community structure (Guo et al., 2016; Viver et al., 2020). It is reported here that changes in microbial community structure produced by dilution of rhizosphere saline soil (water-only) and incubation only (without treatment), observable by differences in ecological alpha and beta indices, reverted after 30 days. This resilience effect was not detected when any four ACs were added to microcosms, as reported on non-saline soil contaminated with AC (Rodríguez-Valdecantos et al., 2017). It was found that the specific compound, rather than its concentration, played a significant role in influencing the bacterial community structure in this rhizosphere saline soil, affecting the removal performance of the microbial community (Figures 2, 3). For example, bacterial communities were strongly affected when 2,4-D was amended at high and low concentrations, increasing richness and diversity. As previously reported, such an increase may be explained by the predominance of cooperative interactions (Rodríguez-Valdecantos et al., 2017). 2,4-D was predictably the less degradable of the four ACs tested here, as the presence of halogen substituents makes their removal difficult during biodegradation (Pieper et al., 2010). In contrast, Phe, Tyr, and 4-HB can be degraded by more than one catabolic aerobic and anaerobic pathway (Pieper et al., 2010; Pérez-Pantoja et al., 2010a,b, 2012).

While the literature associated with 2,4-D degradation by halophiles is very scarce (Maltseva et al., 1996), studies on the degradation of Phe and 4-HB in saline conditions are more common, reporting that degradative aerobic metabolic pathways are similar between halophiles and non-halophiles (Bonfá et al., 2013; Veenagayathri and Vasudevan, 2015; Li et al., 2019; Mahmood et al., 2019). Phe has a general bactericidal effect, but many microorganisms have developed mechanisms to protect themselves and to use it as a sole carbon and energy source through the *ortho* and the *meta* ring-cleavage pathways (Pieper et al., 2010; Pérez-Pantoja et al., 2012; Li et al., 2019). For example, Phe is degraded by *Halomonas organivorans* (Bonfá et al., 2013), and a moderate halophile bacterial consortium, composed of *B. cereus, Arthrobacter* sp., *B. licheniformis, H. salina, B. pumilus,* and *Pseudomonas aeruginosa* (Veenagayathri and Vasudevan, 2015). Our findings are consistent with this as *Pseudomonadota* and *Bacillota* isolates (Figure 4) exhibited significant Phe removal levels.

4-HB is expected to be more easily degraded than Phe (Pérez-Pantoja et al., 2010a,b). Higher removal levels in microcosm experiments confirmed this possibility, as the more significant number of isolates obtained and the higher biomass levels reported here. Tyr is also expected to be readily degradable as Tyr catabolic pathways are frequently found in aerobic bacterial species (Pérez-Pantoja et al., 2010b, 2012), including recent reports demonstrating such degradative capacity in a few *Halomonas* species (Chen et al., 2018; Li et al., 2020), and other halophiles (Dastgheib et al., 2017). Accordingly, halophile organisms belonging to various genera have been shown to degrade hydrocarbons, with members of *Halomonas, Alcanivorax*, *Marinobacter*, *Haloferax*, *Haloarcula*, and *Halobacterium* frequently reported in the literature (Haddadi and Shavandi, 2013; Fathepure, 2014; Dastgheib et al., 2017; Chen et al., 2018; Li et al., 2019, 2020).

The study of AC degradation in microorganisms associated with halophytes, such as members of the family *Amaranthaceae* (Mora-Ruiz et al., 2015; Marasco et al., 2016; Szymańska et al., 2016; Razzaghi Komaresofla et al., 2019), has been less explored and opens possibilities to find new microbial species with catabolic potential. For example, in the *Salicornia europaea* rhizosphere, microbial community composition varies in sites that differ in their salinity level (Szymańska et al., 2016). *Actinomycetota* and *Bacillota* dominate in low salinity sites, whereas *Pseudomonadota* predominates in saline areas with a salinity level like the soil used in our study. Even though a different selective isolation method was used, *Gammaproteobacteria* and *Bacillota* were dominant among the isolates reported here. Members of these two phyla were also described in saline soils and rhizosphere samples associated with six other halophytic plants (Siddikee et al., 2010).

About half of the isolates reported here belong to *Halomonas*, which, among halophiles, possess the highest versatility to metabolize AC (García et al., 2005; Bonfá et al., 2013; Haddadi and Shavandi, 2013). As *Halomonas*, *Marinobacter* species can also degrade AC (Gao et al., 2013), so, unsurprisingly, *Marinobacter* isolates were also found in our study, removing 4-HB, Phe, and Tyr under moderate salinity conditions. Moreover, it has been demonstrated that *Marinobacter hydrocarbonoclasticus* resists high salinity levels without losing degradative activity (Fernandez-Linares et al., 1996). *M. sedimentalis* and *M. falvimaris* isolated from hypersaline sabkhas use biphenyl, phenanthrene, anthracene, and naphthalene as sole carbon and energy sources at moderate salinity (Al-Mailem et al., 2013; Gao et al., 2013).

Several members of the *Bacillaceae* family were also isolated in this work: isolates of *Halobacillus*, *Oceanobacillus, Thalassobacillus,* and *Alkalihalobacillus* grew with Phe and Tyr, and some of them with 4-HB, which agrees with previous reports (Fernandez-Linares et al., 1996; Siddikee et al., 2010; Al-Mailem et al., 2013; Gao et al., 2013). Finally, the only archaeal isolate reported here, able to grow in three of the four compounds tested at salt concentrations of 4.5%, is a possible new species of *Haladaptatus*, which agrees with the versatility of this archaeal genus (Liu et al., 2015; Acikgoz and Ozcan, 2016).

The study of the interaction between *A. thaliana* and the halophilic microorganisms isolated from the rhizosphere of *A. vaginata* found that the halophile *Halomonas* sp. LV-8T, and *Marinobacter* sp. LV-48T were the most interesting, as they exhibited a growth-promoting and protective effect on *A. thaliana* plants under saline stress conditions. This effect was mild on root length and the number of leaves but significant for the rosette area (Figure 8). These results support previous studies that show plant growth-promoting abilities in bacteria belonging to the same genera as *Halomonas* sp. LV-8T and, *Marinobacter* sp. LV-48T (Yadav and Saxena, 2018).

In contrast to strains LV-8T and LV-48T, the halophiles *Alkalihalobacillus* sp. LV-39H and *Thalassobacillus* sp. LV-41P showed adverse effects on the growth of *A. thaliana* under saline stress alone and with additional exposure to AC (Figure 8). Adverse effects may be explained when isolates interfere with phytohormone turnover or are inoculated in high numbers (Poupin et al., 2013; Zúñiga et al., 2013) These latter two microorganisms degrade AC and have plant growth promotion abilities under saline stress conditions on salt-sensitive plants (Orhan, 2016; Shah et al., 2017; Khunjamayum et al., 2022). It would be interesting to test combinations of these isolates as they would perform better, promoting plant growth. It is worth mentioning that the four AC differentially affected *A. thaliana* growth parameters with rosette area >> the number of leaves > root length being more discriminative (Figure 8B). In addition, the detrimental effect of 2,4-D on plant growth parameters, compared to the other three AC, was observed (Figure 8B).

These findings are significant because they suggest halophiles could be biofertilizers for plants grown under high salinity conditions. Furthermore, the ability of these halophiles to protect plants from toxic ACs indicates that they could also be used as bio(phyto)remediation agents for contaminated soils. To do so, plants other than *A. thaliana*, more field-related conditions for plant growth tests (artificial soils, among others), and isolates from other halophyte’s rhizospheres should be used.

## Data availability statement

The datasets presented in this study can be found in online repositories. The names of the repository/repositories and accession number(s) can be found in the article/supplementary material.

## Author contributions

GR-V and FT-R performed most of the work on isolation and initial characterization of halophiles. GR-V performed soil microcosms studies. FT-R performed HPLC analyses. TL and SM-E carried out plant-microbe interactions studies. MM-R and RR-M prepared and analyzed phylogenetic tree. LC-C helped with further characterization of isolates, and microcosms studies. RR-M and BG conceived the whole idea, guided young researchers and provide funding. GR-V, FT-R, SM-E, MM-R, RR-M, LC-C, TL, and BG helped with draft writing. BG prepared and revised final manuscript. All authors contributed to the article and approved the submitted version.

## References

[ref1] Acikgoz E. Ozcan B. (2016). Phenol biodegradation by halophilic archaea. Int. Biodeterior. Biodegrad. 107, 140–146. doi: 10.1016/j.ibiod.2015.11.016

[ref2] Al Farraj D. A. Hadibarata T. Yuniarto A. Alkufeidy R. M. Alshammari M. K. Syafiuddin A. (2020). Exploring the potential of halotolerant bacteria for biodegradation of polycyclic aromatic hydrocarbon. Bioprocess Biosyst. Eng. 43, 2305–2314. doi: 10.1007/s00449-020-02415-4, PMID: 32812060

[ref3] Al-Mailem D. M. Eliyas M. Radwan S. S. (2013). Oil-bioremediation potential of two hydrocarbonoclastic, diazotrophic *Marinobacter* strains from hypersaline areas along the Arabian gulf coasts. Extremophiles 17, 463–470. doi: 10.1007/s00792-013-0530-z, PMID: 23543287

[ref4] Álvarez H. M. Silva R. (2013). “Metabolic diversity and flexibility for hydrocarbon biodegradation by *Rhodococcus*” in Actinobacteria: application in bioremediation and production of industrial enzymes. eds. Amoroso M. J. Benimeli C. S. Cuozzo S. A.. 1st ed (Boca Raton, FL: CRC Press), 241–273.

[ref5] Aracil-Gisbert S. Torregrosa-Crespo J. Martínez-Espinosa R. M. (2018). “Recent trends on bioremediation of polluted salty soils and waters using *Haloarchaea*” in Advances in bioremediation and phytoremediation. ed. Shiomi N. (London: IntechOpen), 63–77.

[ref6] Arora S. Singh A. Singh Y. (2017). “Microbial approach for bioremediation of saline and sodic soils” in Bioremediation of salt affected soils: an Indian perspective. eds. Arora S. Singh A. Singh Y. (Cham: Springer), 87–100.

[ref7] Blackwood C. B. Hudleston D. Zak D. R. Buyer J. S. (2007). Interpreting ecological diversity indices applied to terminal restriction fragment length polymorphism data: insights from simulated microbial communities. Appl. Environ. Microbiol. 73, 5276–5283. doi: 10.1128/AEM.00514-07, PMID: 17601815 PMC1950973

[ref8] Bonfá M. R. L. Grossman M. J. Piubeli F. Mellado E. Durrant L. R. (2013). Phenol degradation by halophilic bacteria isolated from hypersaline environments. Biodegradation 24, 699–709. doi: 10.1007/s10532-012-9617-y, PMID: 23292008

[ref9] Campbell B. J. Kirchman D. L. (2013). Bacterial diversity, community structure and potential growth rates along an estuarine salinity gradient. ISME J. 7, 210–220. doi: 10.1038/ismej.2012.93, PMID: 22895159 PMC3526181

[ref10] Chaudhary P. Xu M. Ahamad L. Chaudhary A. Kumar G. Saanu Adeleke B.. (2023). Application of synthetic consortia for improvement of soil fertility, pollution remediation, and agricultural productivity: a review. Agronomy 13:643. doi: 10.3390/agronomy13030643

[ref11] Chen C. Anwar N. Wu C. Fu G. Wang R. Zhang C.. (2018). *Halomonas endophytica* sp. nov., isolated from liquid in the stems of *Populus euphratica*. Int. J. Syst. Evol. Microbiol. 68, 1633–1638. doi: 10.1099/ijsem.0.002585, PMID: 29561252

[ref12] Clarke K. (1993). Nonparametric multivariate analyses of changes in community structure. Austral Ecol. 18, 117–143. doi: 10.1111/j.1442-9993.1993.tb00438.x

[ref13] Dastgheib S. M. M. Tirandaz H. Nikou M. M. Ramezani M. Shavandi M. Amoozegar M. A.. (2017). *Prauserella oleivorans* sp. nov., a halophilic and thermotolerant crude-oil-degrading actinobacterium isolated from an oil-contaminated mud pit. Int. J. Syst. Evol. Microbiol. 67, 3381–3386. doi: 10.1099/ijsem.0.002124, PMID: 28857021

[ref14] Elkin L. A. Kay M. Higgins J. J. Wobbrock J. O. (2021). An aligned rank transform procedure for multifactor contrast tests. The 34th Annual ACM Symposium on User Interface Software and Technology

[ref16] Fathepure B. Z. (2014). Recent studies in microbial degradation of petroleum hydrocarbons in hypersaline environments. Front. Microbiol. 5:173. doi: 10.3389/fmicb.2014.00173, PMID: 24795705 PMC4005966

[ref17] Fernandez-Linares L. Acquaviva M. Bertrand J. C. Gauthier M. (1996). Effect of sodium chloride concentration on growth and degradation of eicosane by the marine halotolerant bacterium *Marinobacter hydrocarbonoclasticus*. Syst. Appl. Microbiol. 19, 113–121. doi: 10.1016/S0723-2020(96)80018-X

[ref18] Gao W. Cui Z. Li Q. Xu G. Jia X. Zheng L. (2013). *Marinobacter nanhaiticus* sp. nov., polycyclic aromatic hydrocarbon-degrading bacterium isolated from the sediment of the South China Sea. Antonie Van Leeuwenhoek 103, 485–491. doi: 10.1007/s10482-012-9830-z, PMID: 23117603

[ref9001] García M. T. Ventosa A. Mellado E. (2005). Catabolic versatility of aromatic compound-degrading halophilic bacteria. FEMS Microbiol. Ecol. 54, 97–109. doi: 10.1016/j.femsec.2005.03.009 16329976

[ref19] Guo G. He F. Tian F. Huang Y. Wang H. (2016). Effect of salt contents on enzymatic activities and halophilic microbial community structure during phenanthrene degradation. Int. Biodeterior. Biodegrad. 110, 8–15. doi: 10.1016/j.ibiod.2016.02.007

[ref20] Haddadi A. Shavandi M. (2013). Biodegradation of phenol in hypersaline conditions by *Halomonas* sp. strain PH2-2 isolated from saline soil. Int. Biodeterior. Biodegrad. 85, 29–34. doi: 10.1016/j.ibiod.2013.06.005

[ref21] Honda M. Suzuki N. (2020). Toxicities of polyciclic aromatic hydrocarbons for aquatic animals. Int. J. Environ. Res. Public Health 17:1363. doi: 10.3390/ijerph17041363, PMID: 32093224 PMC7068426

[ref22] Hothorn T. Bretz F. Westfall P. (2008). Simultaneous inference in general parametric models. Biom. J. 50, 346–363. doi: 10.1002/bimj.200810425 18481363

[ref23] Jafari B. Hanifezadeh M. Parvin M. S. J. (2012). Molecular study of bacteria associated with Salicornia symbiotic bacteria as a candidate for Hormozgan salty zone culturing by Persian gulf water irrigation. Afr. Microbiol. Res. 6, 4687–4695. doi: 10.5897/AJMR11.1132

[ref24] Kapadia C. Patel N. Rana A. Vaidya H. Alfarraj S. Ansari M. J.. (2022). Evaluation of plant growth-promoting and salinity ameliorating potential of halophilic bacteria isolated from saline soil. Front. Plant Sci. 13:946217. doi: 10.3389/fpls.2022.946217, PMID: 35909789 PMC9335293

[ref25] Khunjamayum R. Tamreihao K. Asem R. S. Singh Y. R. Nongthombam A. Devi K. M.. (2022). Fungal disease suppression and growth promotion potential of endophytic bacteria from ethnomedicinal plants. Arch. Microbiol. 204:539. doi: 10.1007/s00203-022-03136-w, PMID: 35927385

[ref26] Kimbrel J. A. Ballor N. Wu Y.-W. David M. M. Hazen T. C. Simmons B. A.. (2018). Microbial community structure and functional potential along a hypersaline gradient. Front. Microbiol. 9:1492. doi: 10.3389/fmicb.2018.01492, PMID: 30042744 PMC6048260

[ref27] Krzyszczak A. Czech B. (2021). Ocurrence and toxicity of polyciclic aromatic hydrocarbons derivatives in environmental matrices. Sci. Total Environ. 788:147738. doi: 10.1016/j.scitotenv.2021.147738, PMID: 34023603

[ref28] Lenth R. Singmann H. Love J. Buerkner P. Herve M. (2018). Package Emmeans. R package version 4.0.3. (Comprehensive R Archive Network). R Core Team. Available at: http://cran.r-project.org/package=emmeans

[ref29] Li X. Gan L. Hu M. Wang S. Tian Y. Shi B. (2020). *Halomonas pellis* sp. nov., a moderately halophilic bacterium isolated from wetsalted hides. Int. J. Syst. Evol. Microbiol. 70, 5417–5424. doi: 10.1099/ijsem.0.004426, PMID: 32886591

[ref30] Li H. Meng F. Duan W. Lin Y. Zheng Y. (2019). Biodegradation of phenol in saline or hypersaline environments by bacteria: a review. Ecotoxicol. Environ. Saf. 184:109658. doi: 10.1016/j.ecoenv.2019.109658 31520955

[ref31] Liu G. Zhang M. Mo T. He L. Zhang W. Yu Y.. (2015). Metabolic flux analysis of the halophilic archaeon *Haladaptatus paucihalophilus*. Biochem. Biophys. Res. Commun. 467, 1058–1062. doi: 10.1016/j.bbrc.2015.09.174, PMID: 26441084

[ref32] Ma Y. Galinski E. A. Grant W. D. Oren A. Ventosa A. (2010). Halophiles 2010: life in saline environments. Appl. Environ. Microbiol. 76, 6971–6981. doi: 10.1128/AEM.01868-10, PMID: 20817804 PMC2976238

[ref33] Mahmood A. Kataoka R. Turgay O. C. Yaprak A. E. (2019). “Halophytic microbiome in ameliorating the stress” in Ecophysiology, abiotic stress responses and utilization of halophytes. eds. Hasanuzzaman M. Nahar K. Öztürk M. (Singapore: Springer), 171–194.

[ref34] Mainka T. Weirathmüller D. Herwig C. Pflügl S. (2021). Potential applications of halophilic microorganisms for biological treatment of industrial process brines contaminated with aromatics. J. Ind. Microbiol. Biotechnol. 48:kuab015. doi: 10.1093/jimb/kuab015, PMID: 33928348 PMC9113102

[ref35] Mallick I. Bhattacharyya C. Mukherji S. Dey D. Sarkar S. C. Mukhopadhyay U. K.. (2018). Effective rhizoinoculation and biofilm formation by arsenic immobilizing halophilic plant growth promoting bacteria (PGPB) isolated from mangrove rhizosphere: a step towards arsenic rhizoremediation. Sci. Total Environ. 610-611, 1239–1250. doi: 10.1016/j.scitotenv.2017.07.234, PMID: 28851144

[ref36] Maltseva O. McGowan C. Fulthorpe R. Oriel P. (1996). Degradation of 2,4-dichlorophenoxyacetic acid by haloalkaliphilic bacteria. Microbiology 142, 1115–1122. doi: 10.1099/13500872-142-5-1115, PMID: 8704953

[ref37] Marasco R. Mapelli F. Rolli E. Mosqueira M. J. Fusi M. Bariselli P.. (2016). *Salicornia strobilacea* (synonym of *Halocnemum strobilaceum*) grown under different tidal regimes selects rhizosphere bacteria capable of promoting plant growth. Front. Microbiol. 7:1286. doi: 10.3389/fmicb.2016.01286, PMID: 27597846 PMC4992691

[ref38] Molina L. Segura A. (2021). Biochemical and metabolic plant responses towards polyciclic aromatic hydrocarbons and heavy metals present in atmospheric pollution. Plan. Theory 10:2305. doi: 10.3390/plants10112305, PMID: 34834668 PMC8622723

[ref39] Mora-Ruiz M. R. Font-Verdera F. Díaz-Gil C. Urdiain M. Rodríguez-Valdecantos G. González B.. (2015). Moderate halophilic bacteria colonizing the phylloplane of halophytes of the subfamily *Salicornioideae* (*Amaranthaceae*). Syst. Appl. Microbiol. 38, 406–416. doi: 10.1016/j.syapm.2015.05.004, PMID: 26164126

[ref40] Murashige T. Skoog F. (1962). A revised medium for rapid growth and bioassays with tobacco tissue cultures. Physiol. Plant. 15, 473–497. doi: 10.1111/j.1399-3054.1962.tb08052.x

[ref41] Ning X. Shen L. Sun J. Lin C. Zhang Y. Yang Z.. (2015). Degradation of polycyclic aromatic hydrocarbons (PAHs) in textile dyeing sludge by O_3_/H_2_O_2_ treatment. RSC Adv. 5, 38021–38029. doi: 10.1039/C5RA03307A

[ref42] Oksanen J. Blanchet F. G. Friendly M. Kindt R. Legendre P. Mc Glinn D.. (2020). Vegan: community ecology package. R package version 2, 5–7. Available at: https://CRAN.R-project.org/package=vegan

[ref43] Orhan F. (2016). Alleviation of salt stress by halotolerant and halophilic plant growth-promoting bacteria in wheat (*Triticum aestivum*). Braz. J Microbiol. 47, 621–627. doi: 10.1016/j.bjm.2016.04.001 27133557 PMC4927673

[ref44] Osborne C. A. Rees G. N. Bernstein Y. Janssen P. H. (2006). New threshold and confidence estimates for terminal restriction fragment length polymorphism analysis of complex bacterial communities. Appl. Environ. Microbiol. 72, 1270–1278. doi: 10.1128/AEM.72.2.1270-1278.2006, PMID: 16461676 PMC1392978

[ref45] Pérez-Pantoja D. De la Iglesia R. Pieper D. H. González B. (2008). Metabolic reconstruction of aromatic compounds degradation from the genome of the amazing pollutant-degrading bacterium *Cupriavidus necator* JMP134. FEMS Microbiol. Rev. 32, 736–794. doi: 10.1111/j.1574-6976.2008.00122.x, PMID: 18691224

[ref46] Pérez-Pantoja D. Donoso R. Agulló L. Córdova M. Seeger M. Pieper D. H.. (2012). Genomic analysis of aromatic compounds biodegradation in *Burkholderiales*. Environ. Microbiol. 14, 1091–1117. doi: 10.1111/j.1462-2920.2011.02613.x, PMID: 22026719

[ref47] Pérez-Pantoja D. Donoso R. Junca H. González B. Pieper D. H. (2010b). “Phylogenomics of aerobic bacterial degradation of aromatics” in Handbook of hydrocarbon and lipid microbiology. ed. Timmis K. N. (Berlin: Springer-Verlag), 1356–1397.

[ref48] Pérez-Pantoja D. González B. Pieper D. H. (2010a). “Aerobic degradation of aromatic hydrocarbons” in Handbook of hydrocarbon and lipid microbiology. ed. Timmis K. N. (Berlin: Springer), 799–837.

[ref49] Pieper D. H. González B. Cámara B. Pérez-Pantoja D. Reineke W. (2010). “Aerobic degradation of chloroaromatics” in Handbook of hydrocarbon and lipid microbiology. ed. Timmis K. N. (Berlin: Springer), 839–864.

[ref50] Pinedo I. Ledger T. Greve M. Poupin M. J. (2015). *Burkholderia phytofirmans* PsJN induces long-term metabolic and transcriptional changes involved in *A. thaliana* salt tolerance. Front. Plant Sci. 6:466. doi: 10.3389/fpls.2015.00466, PMID: 26157451 PMC4477060

[ref51] Poupin M. J. Timmermann T. Vega A. Zúñiga A. González B. (2013). Effects of the plant growth promoting bacterium *Burkholderia phytofirmans* PsJN throughout the life cycle of *Arabidopsis thaliana*. PLoS One 8:2013. doi: 10.1371/journal.pone.0069435, PMID: 23869243 PMC3711820

[ref52] Razzaghi Komaresofla B. Alikhani H. A. Etesami H. Khoshkholgh-Sima N. A. (2019). Improved growth and salinity tolerance of the halophyte *Salicornia* sp. by co-inoculation with endophytic and rhizosphere bacteria. Appl. Soil Ecol. 138, 160–170. doi: 10.1016/j.apsoil.2019.02.022

[ref53] Robertson G. P. Coleman D. C. Sollins P. Bledsoe C. S., (1999). Standard soil methods for long-term ecological research New York. Oxford University Press

[ref54] Rodríguez-Valdecantos G. Manzano M. Sánchez R. Urbina F. Hengst M. B. Lardies M. A.. (2017). Early successional patterns of bacterial communities in soil microcosms reveal changes in bacterial community composition and network architecture, depending on the successional condition. Appl. Soil Ecol. 120, 44–54. doi: 10.1016/j.apsoil.2017.07.015

[ref55] RStudio Team. (2020). RStudio: integrated development for R. RStudio PBC, Boston, MA. Available at: http://www.rstudio.com/

[ref56] Sáenz-Mata J. Palacio-Rodríguez R. Sánchez-Galván H. Balagurusamy N. (2016). “Plant growth promoting rhizobacteria associated to halophytes: potential applications in agriculture” in Sabkha ecosystems. Tasks for vegetation science. eds. Khan M. Boër B. Ȫzturk M. Clüsener-Godt M. Gul B. Breckle S. W. (Cham: Springer), 411–425.

[ref9002] Sahu P. K. Shafi Z. Singh S. Ojha K. Jayalakshmi K. Tilgam J.. (2023). Colonization potential of endophytes from halophytic plants growing in the “Runn of Kutch” salt marshes and their contribution to mitigating salt stress in tomato cultivation. Front. Microbiol. 14:1226149. doi: 10.3389/fmicb.2023.1226149 37705729 PMC10495581

[ref57] Sampedro I. Pérez-Mendoza D. Toral L. Palacios E. Arriagada C. Llamas I. (2020). Effects of halophyte root exudates and their components on chemotaxis, biofilm formation and colonization of the halophilic bacterium *Halomonas anticariensis* FP35(T). Microorganisms 8:575. doi: 10.3390/microorganisms8040575, PMID: 32316222 PMC7232322

[ref58] Sardar H. Khalid Z. Ahsan M. Naz S. Nawaz A. Ahmad R.. (2023). Enhancement of salinity stress tolerance in lettuce (*Lactuca sativa* L.) via foliar application of nitric oxide. Plan. Theory 12:1115. doi: 10.3390/plants12051115, PMID: 36903975 PMC10005404

[ref59] Shah G. Jan M. Afreen M. Anees M. Rehman S. Daud M. K.. (2017). Halophilic bacteria mediated phytoremediation of salt—affected soils cultivated with rice. J. Geochem. Explor. 174, 59–65. doi: 10.1016/j.gexplo.2016.03.011

[ref60] Shannon C. E. (1948). A mathematical theory of communication. Bell Syst. Tech J. 27, 623–656. doi: 10.1002/j.1538-7305.1948.tb01338.x

[ref61] Siddikee M. A. Chauhan P. S. Anandham R. Han G. H. Sa T. M. (2010). Isolation, characterization, and use for plant growth promotion under salt stress, of ACC deaminase-producing halotolerant bacteria derived from coastal soil. J. Microbiol. Biotechnol. 20, 1577–1584. doi: 10.4014/jmb.1007.07011, PMID: 21124065

[ref62] Singh B. K. Nazaries L. Munro S. Anderson I. C. Campbell C. D. (2006). Use of multiplex terminal restriction fragment length polymorphism for rapid and simultaneous analysis of different components of the soil microbial community. Appl. Environ. Microbiol. 72, 7278–7285. doi: 10.1128/AEM.00510-06, PMID: 16936053 PMC1636152

[ref63] Szymańska S. Płociniczak T. Piotrowska-Seget Z. Hrynkiewicz K. (2016). Endophytic and rhizosphere bacteria associated with the roots of the halophyte *Salicornia europaea* L.—community structure and metabolic potential. Microbiol. Res. 192, 37–51. doi: 10.1016/j.micres.2016.05.012, PMID: 27664722

[ref64] Veenagayathri K. Vasudevan N. (2015). Degradation of dual phenolics by a moderately halophilic bacterial consortium and its degradation products. Int. J. Curr. Microbiol. App. Sci. 3, 513–524. doi: 10.9734/BMRJ/2013/3171

[ref65] Ventosa A. Quesada E. Rodriguez-Valera F. Ruiz-Berraquero F. Ramos-Cormenzana A. (1982). Numerical taxonomy of moderately halophilic Gram-negative rods. Microbiology 128, 1959–1968. doi: 10.1099/00221287-128-9-1959

[ref66] Viver T. Conrad R. E. Orellana L. H. Urdiain M. González-Pastor J. E. Hatt J. K.. (2020). Distinct ecotypes within a natural haloarchaeal population enable adaptation to changing environmental conditions without causing population sweeps. ISME J. 15, 1178–1191. doi: 10.1038/s41396-020-00842-5, PMID: 33342997 PMC8182817

[ref67] Waddell E. J. Elliott T. J. Vahrenkamp J. M. Roggenthen W. M. Sani R. K. Anderson C. M.. (2010). Phylogenetic evidence of noteworthy microflora from the subsurface of the former Homestake gold mine, Lead, South Dakota. Environ. Technol. 31, 979–991. doi: 10.1080/09593331003789511, PMID: 20662386 PMC3565620

[ref68] Warren-Vega W. M. Campos-Rodríguez A. C. Zárate-Guzmán A. I. Romero-Cano L. A. (2023). A current review of water pollutants in American continent: trends and perspectives in detection, health risks and treatment technologies. Int. J. Environ. Res. Public Health 20:4099. doi: 10.3390/ijerph20054499, PMID: 36901509 PMC10001968

[ref69] Witzgall K. Vidal A. Schubert D. I. Höschen C. Schweizer S. A. Buegger F.. (2021). Particulate organic matter as a functional soil componente for persistent soil organic carbon. Nat. Commun. 12:4115. doi: 10.1038/s41467-021-24192-8, PMID: 34226560 PMC8257601

[ref70] Wobbrock J. O. Findlater L. Gergle D. Higgins J. J. (2011). The aligned rank transform for nonparametric factorial analyses using only ANOVA procedures. Proceedings of the SIGCHI Conference on Human Factors in Computing Systems. pp. 143–146.

[ref9003] Yadav A. N. Saxena A. K. (2018). Biodiversity and biotechnological applications of halophilic microbes for sustainable agriculture. J. Appl. Biol. Biotechnol. 6, 48–55. doi: 10.7324/JABB.2018.60109

[ref71] Zúñiga A. Poupin M. J. Donoso R. A. Ledger T. Guiliani N. Gutiérrez R. A.. (2013). Quorum sensing and indole-3-acetic acid degradation play a role in colonization and plant growth promotion of *Arabidopsis thaliana* by *Burkholderia phytofirmans* PsJN. Mol. Plant Microbe. Interact. 26, 546–553. doi: 10.1094/MPMI-10-12-0241-R, PMID: 23301615

